# Pioneer Role of Extracellular Vesicles as Modulators of Cancer Initiation in Progression, Drug Therapy, and Vaccine Prospects

**DOI:** 10.3390/cells11030490

**Published:** 2022-01-31

**Authors:** Sadaf Jahan, Shouvik Mukherjee, Shaheen Ali, Urvashi Bhardwaj, Ranjay Kumar Choudhary, Santhanaraj Balakrishnan, Asma Naseem, Shabir Ahmad Mir, Saeed Banawas, Mohammed Alaidarous, Hadeel Alyenbaawi, Danish Iqbal, Arif Jamal Siddiqui

**Affiliations:** 1 Department of Medical Laboratory Sciences, College of Applied Medical Sciences, Majmaah University, Al-Majmaah 11952, Saudi Arabia; r.choudhary@mu.edu.sa (R.K.C.); a.naseem@mu.edu.sa (A.N.); s.mir@mu.edu.sa (S.A.M.); s.banawas@mu.edu.sa (S.B.); m.alaidarous@mu.edu.sa (M.A.); hadeel.a@mu.edu.sa (H.A.); da.mohammed@mu.edu.sa (D.I.); 2 Department of Biotechnology, School of Chemical and Life Sciences, Jamia Hamdard, Hamdard Nagar, New Delhi 110062, India; shvkmkhrj@gmail.com (S.M.); yasmeen123shaheen@gmail.com (S.A.); urvashibhardwaj97@gmail.com (U.B.); 3 Medical Equipment Technology, College of Applied Medical Sciences, Majmaah University, Al-Majmaah 11952, Saudi Arabia; s.balakrishnan@mu.edu.sa; 4 Department of Biomedical Sciences, Oregon State University, Corvallis, OR 97331, USA; 5 Department of Biology, College of Science, University of Hail, Hail 81451, Saudi Arabia; arifjamal13@gmail.com

**Keywords:** cancer, extracellular vesicles, diagnostic tools, long non-coding RNAs, miRNA, angiogenesis, immune response, metastasis, exosomes

## Abstract

Cancer is one of the leading diseases, causing deaths worldwide. Nearly 10 million deaths were reported in 2020 due to cancer alone. Several factors are involved in cancer progressions, such as lifestyle and genetic characteristics. According to a recent report, extracellular vesicles (EVs) are involved in cancer initiation, progression, and therapy failure. EVs can play a major role in intracellular communication, the maintenance of tissue homeostasis, and pathogenesis in several types of diseases. In a healthy person, EVs carry different cargoes, such as miRNA, lncRNA etc., to help other body functions. On the other hand, the same EV in a tumor microenvironment carries cargoes such as miRNA, lncRNA, etc., to initiate or help cancer progression at various stages. These stages may include the proliferation of cells and escape from apoptosis, angiogenesis, cell invasion, and metastasis, reprogramming energy metabolism, evasion of the immune response, and transfer of mutations. Tumor-derived EVs manipulate by altering normal functions of the body and affect the epigenetics of normal cells by limiting the genetic makeup through transferring mutations, histone modifications, etc. Tumor-derived EVs also pose therapy resistance through transferring drug efflux pumps and posing multiple drug resistances. Such EVs can also help as biomarkers for different cancer types and stages, which ultimately help with cancer diagnosis at early stages. In this review, we will shed light on EVs’ role in performing normal functions of the body and their position in different hallmarks of cancer, in altering the genetics of a normal cell in a tumor microenvironment, and their role in therapy resistance, as well as the importance of EVs as diagnostic tools.

## 1. Introduction

Cancer is a disease in which the body’s cells grow uncontrollably and spread throughout. Normally, cells replicate, grow according to the body’s need, and either die with the aging process or are not needed due to damage [1]. On the other hand, cancer cells keep proliferating and evade apoptosis. When a tumor develops, it can be confined to the primary location and does not spread to another area (called benign). However, some cancer cells can evade and move to other tissues away from their primary site, and such tumors are called malignant. The process by which cancer cells invade other body sites is called metastasis [2]. Before a normal cell becomes cancerous, the cell undergoes a series of transformations. These transformations are (1) dysplasia, (2) hyperplasia, and (3) carcinoma in situ. Cells grow faster and accumulate on site in dysplasia, which, as per the National Institute of Cancer, is a term used to describe the presence of abnormal cells within a tissue or organ. Dysplasia is not cancer, but it may sometimes become cancer. Dysplasia can be mild, moderate, or severe, depending on how abnormal the cells look under a microscope and how much of the tissue or organ is affected. Hyperplasia is a more advanced form of dysplasia. In hyperplasia, there is an increase in the number of cells in an organ or tissue. These cells appear normal under a microscope. They are not cancer but may become cancer. The last transformation is carcinoma in situ. It is the most advanced form that occurs before metastasis. Carcinoma in situ is a clump of abnormal cells that will not migrate beyond the site where they first developed, but they may subsequently spread into normal tissue and cause cancer. Therefore, they are also called Stage-0 diseases. The distinction between dysplasia and carcinoma in situ, in many cases, is difficult. The most advanced form that occurs before metastasis occurs is infiltrating carcinoma. Some tumors can metastasize and others do not. Carcinoma in situ has higher chances to evolve and become actual cancer by metastasis [3]. Some of the main unique characteristics of cancer cells are: (a) they may grow in the absence of signals; (b) as mentioned earlier, they can evade programmed cell death or apoptosis; (c) they may spread to other parts of the body by invading adjacent regions (when normal cells contact other cells, they cease growing, and most normal cells do not migrate across the body. Such a condition is also termed epithelial to mesenchymal transition, where cancer cells move to other parts of the body); (d) they direct blood vessels to grow towards tumors for nutrient supply—this is also called angiogenesis; (e) they can evade the immune response—in fact, sometimes they can trick the immune system into helping them grow and stay alive; (f) can they alter the number of chromosomes, causing cell mutation, thereby changing their genetic composition; (g) they rely on nutrients that are different from normal cells [4]. Cancer can be of different types; there have been more than 200 types of cancer reported. Cancers can also be classified based on the type of cell involved, such as epithelial or squamous cells. Some of the most common types of cancer (based on a specific type of cells) are carcinomas, sarcoma, leukemia, lymphoma, multiple myeloma, melanoma, brain, and spinal cord cancers [5].

In recent years, more research has demonstrated the role of extracellular vesicles (EVs) in cancers. EVs are small cell components that can transport various kinds of bioactive molecules. They carry cargo that is involved in various signaling pathways, which are important in preventing diseases [6]. Despite their importance, a recent paper, focused on cancer, reviewed the interesting role of EVs’ cargo in cancer cell proliferation by altering pathways, escaping apoptosis, promoting metastasis, aiding angiogenesis, evading immune responses inducing therapy resistance, etc. [7]. Some clinical research revealed that EVs are a diagnostic tool as biomarkers and that they are useful for cancer therapy [8,9,10]. In addition, some research also showed the possible role of EVs in altering the genetic content in the tumor microenvironment [11,12]. This review will provide an overview of EVs and their role in normal and tumor microenvironment conditions. Furthermore, we discussed the role of (tumor-derived) EVs in various hallmarks of cancer, altering the genetic content in the tumor microenvironment (TME). The possible ways and new dimensions in which EVs can be used in the diagnosis and treatment of cancer will also be discussed in this review. In addition, the history of exosomes, their role in disease (cancer) progression and diagnosis, as well as their therapeutic efficacy, will also be highlighted here.

## 2. Extracellular Vesicles (EVs)

### 2.1. History, Functions, and Size of EVs

EVs are lipid bilayer vesicles released by cells into the extracellular environment [13]. They are small cell components that can exclusively transport bioactive molecules. To understand the history of EVs, we need to look three decades back. In reticulocytes, the transferrin receptors are associated with small vesicles that are approximately 50 nm (nanometer) in size. It was reported, around 30 years ago, in two 1983 papers by Clifford Harding and Philip Stahl in *JCB* and by Bin-Tao Pan and Rose M. Johnstone in *Cell* [14,15], published within a week of each other. The name “exosome” was kept later by Rose Johnstone, but the term was used earlier while referring to other membrane fragments that are isolated from biological fluids [16,17]. In the year 1997, a paper published by Philip Mitchel and his team from the European Molecular Biology Laboratory (Heidelberg, Germany) used the term “exosome complex”, but for a very different context, as it was meant for intracellular particles involved in RNA editing [18]. Later the nomenclature “exosomes” was adapted for 40–100 nm particles released during reticulocyte differentiation as a result of multivesicular endosome fusion with the plasma membrane [19,20]. A decade later, exosomes were found to be released by B-lymphocytes and dendritic cells in a similar way [21,22]. Later, it was found that, in cytotoxic T cells, platelets, mast cells, neurons, oligodendrocytes, Schwann cells, and intestinal epithelial cells that released exosomes in a similar fashion [23,24]. EVs have a size range of 30–1000 nm. Based on their cellular origin or biogenesis, EVs can be divided into exosomes, microvesicles, apoptotic bodies, and large oncosomes [25]. The EVs can operate locally by altering the behavior of adjacent cells, or they can function systemically by travelling via the bloodstream or lymphatic system and impacting cells far away [13].

EVs can be employed as biomarkers because of their molecular composition in the blood or other bodily fluids, which can offer information about their tissue of origin. In addition, they can be utilized to transport and deliver therapeutic molecules because they can target specific tissues and be taken up by specific cells [9]. The specific functions of EVs depend on the cell source and origin.

### 2.2. Cell Source of EVs

EVs have been identified in prokaryotes and eukaryotes from the last few decades as effective intercellular communication carriers. This is due to their ability to transport metabolites, proteins, and nucleic acids, impacting both the recipient and parent cells’ physiological and pathological activities. Some EVs are formed through outward budding or fission. For example, microvesicles, a type of EVs, are formed by the outward budding and fission of the plasma membrane [9]. They originate from vesicles shed by the plasma membrane and are pinched off directly from the plasma membrane [26,27]. Microvesicles have a diameter of 200–1000 nm [28], with a density of 1.04–1.07 g/mL, while some are below 50 nm in diameter, having a density of 1.10–1.18 g/mL [29]. Another type of EVs, called exosomes, are formed within the endosomal network further released with multivesicular bodies of the plasma membrane [28]. The exosomes are intraluminal vesicles (ILVs) that are discharged into the extracellular environment when multivesicular bodies (MVBs) fuse with the plasma membrane [26]. Exosomes (also called small EVs or sEVs) have a diameter of 30–150 nm and a density of 1.13–1.19 g/mL [28]. The third type of EVs, called apoptotic vesicles, are released as blebs of cells that undergo apoptosis [30]. When the plasma membrane blebs during apoptosis, apoptotic bodies are discharged, or put simply, are derived from cells undergoing apoptosis [26]. Apoptotic bodies/vesicles range from 100–1000 nm, 1000–5000 nm, and they have densities ranging from 1.16–1.28 g/mL [29]. Large oncosomes are released by tumor cells and originate from plasma membrane shed vesicles [31]. They have a diameter of 500–4000 nm or 1000–10000 nm, and their density varies from 1.10–1.15 g/mL. EVs serve a core role in the pathogenesis of diseases and tissue homeostasis.

Different cells release EVs that carry some bioactive component to the recipient cells from one cell [26]. Macrophages, intestinal epithelial cells, fibroblasts, mast cells, platelets, apoptotic lymphocytes, cells of the thymus, etc., all secrete EVs that carry cargoes which aid with different functions [32,33,34]. More detailed, but still not exhaustive, information of the source of EVs and EV cargoes, with their specific functions, are summarized in Table 1.

## 3. Intracellular Transfer of Traits between Microenvironment and Tumor Cells

Two mechanisms promote tumor development and progression: genetic/epigenetic alterations in tumor cells, and the reconfiguration of the constituents of the tumor microenvironment via reciprocal and dynamic interaction [52,53]. As the center of the tumor microenvironment, tumor cells employ sophisticated signaling networks to influence the function of cellular and non-cellular components to use non-malignant cells to their advantage [54]. The result of such an interaction is shown in tumor development and upkeep, and in inadequate therapeutic response and multidrug resistance (MDR). In all stages of cancer growth and metastasis, non-malignant cells in the tumor microenvironment are known to endorse tumorigenesis [55,56]. A dense network of cytokines, chemokines, growth factors, inflammatory mediators, and matrix-remodeling enzymes are the source of intercellular communication, but additional and intriguing ways of contact are surfacing. Circulating tumor cells (CTCs), exosomes, cell-free DNA (cfDNA), and apoptotic bodies are examples of new horizontal gene transfer (HGT) mediators which produced from tumor cells and convey information to remote target cells, such as tumor cells and normal cells [54]. The uptake of EVs by recipient cells helps undertake their intercellular communication function. In EV-mediated drug resistance, bidirectional interactions between the tumor microenvironment and tumor cells also play a significant part [57].

## 4. Derived EVs and Epigenetic Biology of EVs

The study of inherited gene-expression modifications without any variations in genotype is known as epigenetics [58]. A variety of pathological diseases, neurological, metabolic, and immunological disorders, and cancer are associated with changes in the epigenetic regulation of gene expression. This refers to modifications to the genome that are functionally relevant but that do not modify the nucleotide sequence while impacting gene expression, ultimately leading to major changes in the tumor microenvironment and influencing various stages of cancer [25,59]. DNA methylation at particular gene promoters, histone modifications, chromatin regulator changes, and miRNA rearrangements are examples of abnormal epigenetic modifications in cancer [60,61]. As a result, these epigenetic abnormalities may contribute to neoplastic tumor transformation and enable cancer immune evasion, metastasis, and both innate and acquired treatment resistance. However, unlike DNA mutations, changes in the epigenome associated with cancer are potentially reversible, which opens up the possibility that “epigenetic drugs” may have a powerful impact on the treatment regimens of various cancers. Below, we have discussed some of the epigenetic changes that EVs impact.

### 4.1. Methylation of DNA Influenced by EVs

Methylation is the addition of a methyl group to the 5-carbon position of cytosine in particular regions of DNA (CpG sites). One of the most ubiquitous mechanisms regulating the transcription of oncogenes and anti-oncogenes is a dynamic change in DNA methylation. DNA methyltransferase 1 (DNMT1) and DNA methyltransferase 3a (DNMT3a) and 3b (DNMT3b) add methyl groups to particular cytosine in regulatory sequences’ CpG islands, thereby silencing them [62]. Genes involved in cell cycle regulation (CDKs), intracellular signaling, DNA repair mechanisms including homologous recombination, nucleotide excision repair, mismatch excision repair, and apoptosis are highly affected by methylation [61,63]. DNA methylation alterations lead to impaired tumor immunogenicity and immune recognition by inhibiting the expression of major molecules required for the interaction of cancer cells with the host’s immune system. These epigenetic changes can potentially impact all aspects of the antigen process, including tumor-associated antigen expression and processing, MHC class I molecule production, and accessory/co-stimulatory surface protein expression [64,65,66]. Furthermore, EVs can promote the malignant transformation of normal cells due to their oncogenic bioactive contents. They can easily modify the methylation status of the genome by inducing certain minor changes in the epigenetic modifications [67].

### 4.2. RNAs and EVs as the Key Players for Cancer Progression

Several studies have confirmed the importance of microRNAs (miRNAs) found in the cargo of EVs on cancer cell proliferation and apoptosis. In the case of leukemia, when EVs from leukemia patients were compared to EVs from healthy donors, they showed a distinct miRNA profile, which indicates the involvement of miRNA in this type of cancer [68]. PTEN (phosphatase and tensin homolog) expression, and its downstream proteins p21 and cyclin D1, are affected by EVs that are shed by esophageal cancer cells, carrying miR-93-5p to recipient cancer cells, which upregulates cell proliferation [69]. EVs carrying miR-1246 in breast cancer cells, which suppresses cyclin-G2 and miR-205, lead to cell proliferation in cholangiocarcinoma. The enhancement of cellular proliferation in colon cancer was attributed to EVs shed by colon cancer cells with high levels of miR-193a and mir-200b. In gastric cancer cells, the cells with EVs carrying miRNA were found to influence cancer cell proliferation via CD97-associated pathways. These included the MAPK (mitogen-activated protein kinase) pathway; the experimental setup used Kyoto Encyclopedia of Genes and Genomes (KEGG) analysis to identify it. Through post-transcriptional suppression of the tumor suppressor gene PDCD4 (programmed cell death 4), EVs carrying miR-21 from ovarian serous carcinoma cells significantly led to malignant transformation and progression [70].

LncRNAs (long non-coding RNAs) are a type of non-coding RNAs. They are around 200 nucleotides long. These RNAs only transcribe, not translate, into proteins. They act as significant regulators of gene expression [71]. LncRNAs can exert epigenetic regulation by recruiting histone-modifying complexes or other regulatory proteins at particular DNA target areas, which can silence or activate gene promoters [72]. For instance, MALAT1 (metastasis-associated lung adenocarcinoma transcript 1) is a cancer-related lncRNA that has been frequently observed. MALAT1 overexpression has been linked to enhanced cell proliferation, invasion, metastasis, apoptosis evasion, a failure in the DNA repair process, and tumor-promoting inflammation; therefore, it can be used in cancer diagnosis and prognosis [71,72,73]. LncRNA’s content in extracellular vesicles may correlate with tumor development, metastasis, and treatment response. TUC339, a long non-coding RNA identified in extracellular vesicles produced from HCC cells (hepatocellular carcinoma cells), has been linked to tumor development, adhesion, and cell cycle progression [74].

LncRNA ZFAS1 carrying EVs released by gastric cancer cells has a significant role in cancer cell proliferation. In an experiment (in vitro), the knockdown of LncRNA ZFAS1 indicated lower cell progression and apoptosis; it also inhibited epithelial to mesenchymal transition, while its presence proved the reverse in gastric cancer, since, it favored cancer cell growth [75]. In colorectal cancer, lncRNA PVT1 has a correlated expression with c-Myc and its regulating genes (NPM1, FUBP1, and EZH2); PVT1 targeted siRNA that transfected SW480 and SW620 cell lines. PVT1 is also correlated with the expression of PVT1-associated transcript nuclear factor κB, as well as myocyte-specific enhancer 2A. Higher expressions of PVT1 also correlated with a higher expression of c-Myc, which is a proto-oncogene; they are functionally responsible for cell growth and escape from apoptosis. EVs secreted by gastric cancer cells contain the lncRNA ZFAS1. When EVs from esophageal cancer patients were compared to EVs from healthy people, the lncRNA ZEB1-AS1 was found at greater levels, indicating a clinical impact [76].

MicroRNAs, functional mRNAs, and lncRNAs, and their regulators, are among the components in the EV cargo that may be implicated in the intercellular transmission of drug resistance [7]. Certain mRNAs mediate the intercellular transmission of drug resistance. DNA methyltransferase 1 (DNMT1) mRNA was found to play a key role in EV-mediated cisplatin resistance in an ovarian cancer xenograft mouse model [77]. Additionally, Glutathione S-transferase P (encoded by GSTP1 gene) an enzyme having capability to detoxify damaging chemicals from affected cells, was transferred to sensitive cells in TME. It is observed that in breast cancer patients having (Adriamycin) resistance to chemotherapy had higher amounts of GSTP1 in comparison to those patients who responded to the therapy, interestingly, through the transfer of miR-122 cargo of EVs secreted by breast cancer cells, changed glucose metabolism in recipient non-tumor cells, facilitating disease progression [7,78].

MicroRNAs are often unregulated during carcinogenesis and metastasis, and drug responses might be affected; certain EV-transferred miRNAs have been linked to drug resistance, as shown in several studies [79]. Moreover, studies on the transport of miRNAs by EVs provided solid evidence to support the development of novel miRNA-targeting methods and the use of miRNAs carried by EVs as predictive indicators of treatment response and drug resistance [80,81]. Following co-culture with MCF7 cells, EVs extracted and purified from a multidrug-resistant chronic myeloid leukemia cell line promoted drug resistance by the EV-mediated transfer of miR-21, which likely resulted in the activation of Akt signaling, thus controlling the NF-B pathway [82]. Furthermore, EVs carrying miR-96 reduced LIM-domain-only protein 7 expressions in lung cancer cells, and EVs transferring miR-222 led to adriamycin resistance in breast cancer cells [83,84].

Additionally, global miRNA profiling of docetaxel-resistant cell lines and the associated EVs revealed a panel of four miRNAs (miR-598, -34-a, -148a, and -146a) that might be used as a diagnostic hallmark for prostate cancer progression [85]. A set of miRNAs was identified exclusively in the EVs of drug-resistant cells, not in the cells themselves, indicating that specific packing into EVs is possible, and miR-1246, -23a, -1469, -638, -1915, -2861, -24, -149star, -3178, -3196, -16, -23b, -762, -663, -26a, -27a, and -1908, as well as let-7a, -7b, and -7c, were the most prominent miRNAs in EVs produced from drug-resistant cells [79]. Other studies have aimed to associate the amounts of certain miRNAs carried by EVs to the acquisition of drug-resistant characteristics in the donor. The expression of miR-221/222, for example, was elevated in drug-resistant breast cancer cells and concentrated in their EVs, and its transfer to drug-sensitive cells was verified [78]. The tamoxifen resistance was transmitted to drug-sensitive cells due to this transfer, which was reversed by anti-miR-221/222 transfection. Therefore, the acquisition of drug-resistant qualities by these cells was also explained by a reduction in the levels of miR-221/222 targets (p27 and estrogen receptor α) in the recipient cells [86].

Recent research concluded that lncRNAs exhibit variable abundance in EVs, similar to what was mentioned earlier for miRNAs, suggesting that these compounds are loaded selectively into EVs. The role of lncRNAs transported by EVs in regulating the CDR phenotype is unknown. Moreover, several researchers have attempted to examine this relationship [87,88]. Through activation of TGF and enhanced expression of ABCG2 (ATP-binding cassette super-family G member 2), the lncRNAs linc-ROR and linc-VLDLR, which were transported by EVs, promoted sorafenib and doxorubicin resistance in hepatocellular carcinoma (HepG2 cells) [89,90]. In addition, after co-culture with HCC-derived EVs, sensitive cells showed an overexpression of linc-VLDR. Chemotherapy-induced cell death was similarly reduced after incubation with these EVs. RNAi-mediated suppression of linc-VLDR in recipient cells resulted in lower cellular survival, as well as lower expressions of ABCG2, indicating the potential of linc-VLDR in drug-resistance promotion [90]. Furthermore, the EV-mediated intercellular transfer of lncARSR enhanced AXL and c-MET expression in renal cell carcinoma cells, which improved sunitinib resistance by competitive interaction with miR-34/miR-449. In addition, lncARSR levels in plasma and tumor tissues were associated with sunitinib response in individuals with renal cell carcinoma [91]. By activating the HMGA I/miR-218 axis, the transfer of lncRNA HOTTIP via EVs enhanced cisplatin resistance in gastric cancer cells. Moreover, high amounts of the EVs’ lncRNA HOTTIP in patient serum were related to a poor cisplatin response during treatment [92].

## 5. Tumor-Derived EVs and Their Approaches in Disease Progression

One of the earliest discovered impacts of EVs in tumors has been promoting or enhancing cell proliferation in recipient cells by transferring oncogenic or tumor suppressor-inhibiting RNAs or proteins or other molecules [93]. EVs with particular cargo that can actively engage in cancer initiation have been related to the release and uptake of many recognized cancer risk factors [94]. Changes in EV release and content have been linked to cancer risk factors, such as environmental chemicals (arsenide or tobacco from smoking cigarettes), diet-related factors (obesity), viral or bacterial infection, hormonal factors (androgen or estrogen), and ultraviolet (UV) radiation, suggesting that EVs may play a role in cancer initiation [9]. Tumor-derived EVs play a crucial role in all stages of cancer development, making EV-based liquid biopsies.

**Figure 1:**
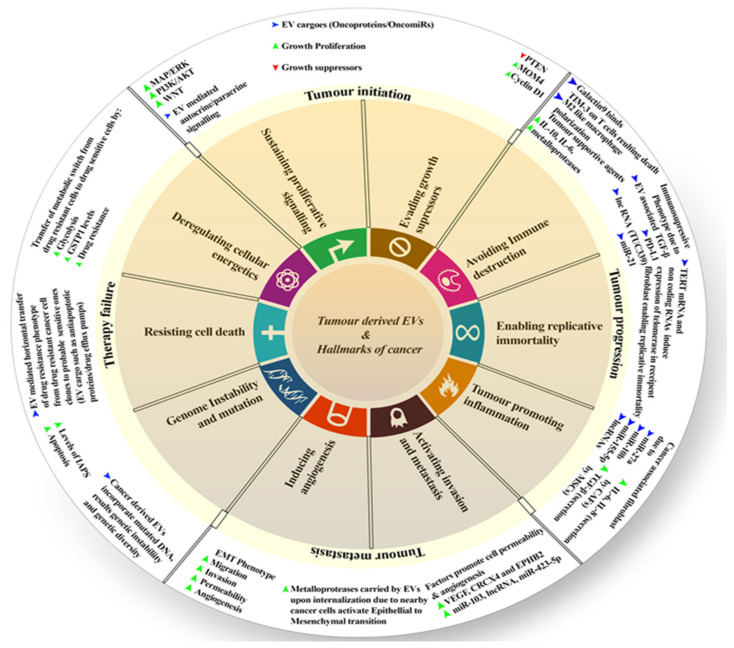
Diagrammatic representation of EVs’ role in influencing different hallmarks of cancer.

Many studies suggested the influence of EVs on cancer hallmarks and their progression, including resistance to treatments. These hallmarks may refer to the changes in cell proliferation and escape apoptosis, angiogenesis, modulating the immune response, finally leading to cell invasion and metastasis, as well as reprogramming the energy metabolism [95]. The typical malignant tumor starts from its initial stages, resulting in cell proliferation and then affecting secondary sites (secondary sites are the sites other than the primary site where the tumor develops). It leads to metastasis, and many cancers at such stages do not respond to therapies, which results in therapy failure. A recent study shows that tumor-derived EVs in the tumor microenvironment have a huge role in cancer initiation, progression, metastasis, and therapy failure (Figure 1). Thus, here, we shall discuss these points.

### 5.1. Initiation

The National Cancer Institute (NCI Dictionary of Cancer Terms) describes tumor initiation as “A process in which normal cells are changed so that they are able to form tumors. Substances that cause cancer can be tumor initiators”. Tumor-derived EVs do play a role in initiation. Such (tumor-derived) EVs support the activation of many tyrosine kinase receptors and downstream signaling pathways, such as MAP/ERK, WNT, PI3K/AKT, etc., leading to proliferative signaling instead of cell suppression. This signaling may be paracrine or autocrine, providing an initial proliferative advantage. Apart from signaling, tumor-derived EVs carry many onco-miRs and oncoproteins. They facilitate further the overriding growth-suppressor signaling, which is achieved by suppressing PTEN and the possible exaggerated expression of cycling D1 and MDM4 (transformed mouse 3T3 cell double minute 4) [95].

### 5.2. Progression

The NCI Dictionary of Cancer Terms describes progression as the course of a disease, such as cancer, as it becomes worse or spreads in the body. EVs do play a great role in tumor progression. IL-10 (interleukin- 10), IL-6 (interleukin- 6), and matrix metalloproteinases (MMPs) are increased in this process. This leads to help with proliferation; all of these are carried by EVs [96,97]. Further, EVs derived from cancer cells serve as vectors for many immunosuppressive agents, such as Galactin-9, that bind to TIM-3 (T cell immunoglobulin and mucin domain-containing protein 3), leading to the death of T cells. When immune cells internalize, EV-associated TGF (transforming growth factor), PD-L1 (programmed death-ligand 1), and many miRs promote an immunosuppressive phenotype. This can involve inducing an M2-like secretion profile in macrophages, in CD8+ T cells, or stimulating B and T cells to produce a variety of tumor-supporting cytokines [98]. TERT (telomerase reverse transcriptase) mRNA and other non-coding RNAs carried by cancer-derived EVs may induce and stimulate telomerase expression in recipient fibroblasts and mutant cell clones, allowing for the development of a cancer stem cell phenotype with replicative immortality. The tumor-derived EVs continue chronic inflammation in the tumor microenvironment. They carry several miRs, such as miR-27, miR-10b, miR-155-5p, and also other non-coding long RNAs. They primarily target fibroblasts and turn them into cancer-associated fibroblasts, which secrete high amounts of TGF-β, IL-6. Some of them (EVs) can also guide the mesenchymal stromal cells (known as MSCs) to secrete IL-8 as well as other possible immunosuppressive cytokines [95].

### 5.3. Metastasis

The NCI Dictionary defines metastasis as “the spread of cancer cells from the place where they first formed to another part of the body. In metastasis, cancer cells break away from the original (primary) tumor, travel through the blood or lymph system, and form a new tumor in other organs or tissues of the body”. Several epigenetic modulators, such as VEGF (vascular endothelial growth factor), CXCR4 (CXC chemokine receptor 4), EPHB2 (EPH Receptor B2), miR-103, or any other sort of long non-coding RNAs, are carried by cancer-derived EVs to stimulate angiogenesis. They improve the permeability of adjacent blood vessels and recruit endothelial tip cells. Many cancer-derived EVs carry MMPs that activate epithelial to mesenchymal transition (EMT), giving the cancer cells mobility. Thus, this induces invasion and metastasis to distant organs. Sometimes, such derived EVs may carry mutated DNA and oncogenes and are heavily dependent upon the primary site of the tumor [95].

### 5.4. Therapy Failure

When mutated DNA is incorporated into a normal cell, it results in genetic instability and diversity. Drug resistance phenotypes can be horizontally transferred from a drug-resistant cancer cell clone to a probable sensitive cancer cell clone via EVs, mediated by possible cargoes such as proteins (anti-apoptotic proteins/drug efflux pumps), lipids, and RNAs (lncRNA/miRNA/mRNA). The same cancer-drug-resistant EVs will cause a metabolic transition in drug-sensitive cancer cells in the recipients. Reprogramming the energy metabolism to glycolysis and boosting the amounts of detoxifying enzymes, such as Glutathione S-transferase P (GSTP1), in these cells results in a multidrug-resistant phenotype [95]. The overall role of EVs as a cancer hallmark is depicted in Figure 1**.** Apart from that, other tumor cell factors involving EVs enhance cancer progression via the modification of normal cells’ function. We will shed light on some factors in the next section.

## 6. Effects of Tumor-Derived EVs on Different Factors Responsible for Cancer Progressions

### 6.1. Role of EVs in Aiding Cell to Escape from Apoptosis

The recruitment of normal cells into the tumorigenic process can be aided by EVs produced by cancer cells. Thus, EVs enhance proliferative signals and promote tumor growth by enabling phenotypic change. Furthermore, EVs can help cancer cells in avoiding apoptosis. RBM11, a splicing factor found in EVs produced by glioblastoma cell lines, was transmitted to recipient tumor cells and caused MDM4 and Cyclin D1 to be spliced into a different oncogenic isoform. It resulted in the cells’ escape apoptosis and increased cell survival ability [99]. It was further proved by a study where EVs from glioblastoma cells were injected into mice, and the ability of the EVs to promote malignancy in recipient tumor cells was verified [99]. In another study related to chloride intracellular channel-1 (CL1C1), EVs released by glioblastoma cells showed facilitated transfer of CL1C1, further supporting the growth of recipient glioblastoma cells. This was further proved by injecting nude mice with glioblastoma cells with EVs containing CL1C1 cargo, it showed enhanced tumor growth in comparison to EVs of glioblastoma cells that did not contain CL1C1. On BRAF mutant (receiver) cells, the transfer of PDGFR (platelet-derived growth factor receptor) mediated by EVs produced by melanoma (donor) cells resulted in activation of the PI3K (phosphoinositide 3 kinases)/Akt pathway and escape from the MAPK pathway; this further helped in enhanced cell growth and escape apoptosis [100].

### 6.2. Impact of EVs on Metastasis from Nearby to Far-Flung Tissues and the Breakdown of the Blood–Brain Barrier

In addition to micro-environmental cells, cancer cells’ EVs can influence other cells in the diverse tumor population, resulting in metastatic capacity transfer. Most cancer cells produce a range of EV types, which influence the activity of receiving cells to the cancer cells’ ultimate advantage. The induction of mesenchymal characteristics in non-tumor cells is a proposed mechanism. It is not only this, but also androgen receptor modulation, TGF- β signaling, and CD151 and CD9 tetraspanins alterations in the recipient cancer cells are how it affects cell invasion and metastasis [101]. Other cancer cell types showed horizontal transfer of the molecules responsible for increasing cellular migration and aiding the invasion of recipient cancer cells mediated by EVs, such as the transfer of Caveolin-1 in breast cancer cells, Wnt5b protein in colon cancer, and CXC chemokine receptor 4 (CXCR4) or SMAD3 in hepatocarcinoma cells [102].

Cancer-derived EVs can impact cells in far-flung tissues, organs, and those closer to the source. EVs appear to be closely linked to brain metastasis as well. The breakdown of the blood–brain barrier is one of the characteristics of brain metastasis. It results in cancer cell migration through it [103]. Tumor cells identify and attach to vascular endothelial membrane components, resulting in extravasation, cancer cell invasion through the Blood brain barrier (BBB), and new growth at secondary organ sites [104]. One such example is EVs that contain miR-181c and that are transferred to brain endothelial cells. Metastatic cells destroy tight junction proteins. Those proteins bind to cytoskeletal protein actin (via decolonizing actin fibers by down-regulating PDPK1 or 3-phosphoinositide–dependent protein kinase–1), including occluding tight junction protein-1 (also called ZO-1) and claudin-5. MiR-105 targets ZO-1 in breast cancer. It is reported that miR-105 and miR-181c are both found in patients with metastatic brain cancer [105].

### 6.3. Reprogramming Energy Metabolism

Tumor cells can change their energy metabolism to fuel their fast cell growth and proliferation. Moreover, there also involves a significant role of EVs in this. In non-tumor cells in the pre-metastatic niche, EV-associated miR-122 from cancer cells downregulates the glycolytic enzyme pyruvate kinase. This downregulation reduces non-tumor cell glucose absorption in the microenvironment. Further, it allows cancer cells in the pre-metastatic niche to obtain more nutrients. In another study, EVs generated from multidrug-resistant leukemia or lung cancer cells caused a metabolic transition in drug-sensitive cancer cells by decreasing the pentose phosphate pathway and, thus, increasing glycolysis. Glutathione S-transferase P1 (GSTP1), an enzyme that can detoxify damaging chemicals from affected cells, was transferred to sensitive cells. In breast cancer, patients with resistance to chemotherapy had higher amounts of GSTP1 than those who responded to the therapy [78].

### 6.4. Angiogenesis

Angiogenesis is the formation of new blood vessels from existing ones. It occurs in both healthy and diseased people throughout one’s life, beginning in the womb and lasting until death. Capillaries are required to exchange nutrients and metabolites in all tissues [106]. Changes in metabolic activity lead to proportional changes in angiogenesis and, hence, proportional changes in capillarity. Oxygen plays a pivotal role in this regulation. Hemodynamic factors are critical for vascular networks’ survival and for the structural adaptations of vessel walls. Endothelial cells aid vascularization by supplying cancer cells with oxygen and nutrients, limited for tumors. The interaction between endothelium and cancer cells is mediated by cytokines such as VEGF (vascular endothelial growth factor) and basic fibroblast growth factor (bFGF).

The cancer cells contain miRNAs and other varieties of molecules that promote angiogenesis [107]. The presence of angiogenic-promoting factors on the cargo of EVs promoted the overexpression of the VEGF on recipient endothelial cells, which aided angiogenesis. These factors include long non-coding RNAs and miRNAs such as lncRNA POU3F3, lncRNA CCAT2, and miR-21, or maybe a receptor-like CXCR4. Cancer cell-derived EV-associated with miR-9 activated the JAK/STAT pathway, which reduced levels of suppressor of cytokine signaling-5 (SOCS5), promoting endothelial cell migration and tumor angiogenesis [108]. Multiple myeloma cells produce EVs that include a piwi-interacting RNA (piRNA-823) that can re-educate endothelial cells to create an environment conducive to the development of multiple myeloma cells. It is accomplished by enhancing interleukin-6, VEGF, and intercellular adhesion molecule-1 expression [109]. MiR-103 in EVs of hepatocarcinoma cells disrupted endothelial cell integrity, leading to angiogenesis. This is done by inhibiting the expression of p20 catenin, VE-cadherin, and zonula occludens-1. EVs containing miR-141-3p in ovarian cancer promoted angiogenesis by activating NF-κB and JAK/STAT3 pathways [110]. Oral cancer cells containing EVs that have miR-142-3p promote angiogenesis mediated by TGFBR1 (transforming growth factor-beta receptor 1). In lung cancer, EVs with miR-23a are suspected of promoting angiogenesis. It also targets testis-specific gene antigen (TSGA 10) in nasopharyngeal carcinoma and promotes angiogenesis. Another most-important miRNA is miR-210, present in cancer cells’ EVs, which promotes angiogenesis by regulating neutral sphingomyelinase-2 [111].

### 6.5. Evasion of Immune Response

Most tumor cells express antigens that allow the immune system to recognize them. However, some cancer cells can avoid the antitumor immune response and, hence, can continue to grow. EVs produced by tumor cells have been widely studied and their effects on many types of neighboring cells in the surrounding TME (tumor microenvironment) engaged in inflammation or immune response [7]. Tumor-derived EVs are high in immune regulatory molecules, including FasL (Fas ligand), TRAIL (TNF-related apoptosis-inducing ligand), and galactin-9, which can help cancer elude the immune system [112]. Fibroblasts, T and B lymphocytes, and macrophages play a huge role in an individual’s immune system [113]. It has been recently reported that tumor-derived EVs have a considerable impact on fibroblasts, T-lymphocytes, B-lymphocytes, and macrophages. In this section, we will briefly discuss tumor-derived EVs’ role in fibroblasts, T-lymphocytes, B-lymphocytes, and macrophages.

#### 6.5.1. Fibroblasts

Interactions between cancer-derived EVs and fibroblasts have been studied well. EVs released by chronic lymphocytic leukemia were incorporated actively near the stromal cells. This induced characteristics of cancer-associated fibroblasts (CAFs), resulting in the secretion of inflammatory cytokines that resulted in a supportive environment for the tumor. In Hodgkin lymphoma cells and ovarian cancer cells, EVs altered fibroblasts’ phenotype, rendering it as a more tumor-supportive environment, which leads to aiding tumor growth and progression [103,114]. EVs in gastric cancer cells modulated pericytes proliferation and migration, which also suggests that it induced the expression of CAF markers in pericytes. For the transformation of normal hepatocytes stellate cells to CAFs, miR-21 found in EVs was found to be responsible for the transformation of normal cells. Clinical study also reveals the presence of miR-21 in higher amounts, which is also correlated with the activation of CAFs and higher cell densities; thus, it may be responsible for hepatocellular carcinoma, enhancing, therefore, tumor development [115]. In metastatic osteosarcoma, miR-675 found in EVs is associated with downregulating CALN1 expression in fibroblast cells (non-malignant), increasing their invasion and migration capabilities. In the presence of EVs produced by pancreatic cancer cell lines, primary pancreatic fibroblasts from mice were transformed into CAF-like cells, a process controlled by miR-155 found in the EVs’ cargo [116]. EVs carrying cargoes like miR-27a in gastric cancer, miR-155-5p in melanoma cells, miR-10b in colorectal cancer, miR-142-3p in lung cancer cells shown to have subsequent influence to transform fibroblasts to CAFs [117].

#### 6.5.2. T lymphocytes

Tumor-derived EVs may potentially affect T cells. To illustrate, glioblastoma cells containing EVs expressed PD-L1, a transmembrane protein that can bind to T cells and block its activation, allowing cancer cells to proliferate. CTLA-4 (cytotoxic T lymphocyte-associated antigen–4) plays an important role in T cell exhaustion and co-inhibition. Additionally, PD-L1 ligand is increased in many malignancies and inhibits cytotoxic T cell activation, which leads to a poor prognosis. CTLA-4 inhibition also inhibits the activation of T effector cells [118,119]. Other examples include EVs containing miR-24-3p by repressing the fibroblast growth factor 11, which aids in T cell dysfunction in nasopharyngeal carcinoma. The CEACAM (called carcinoembryonic antigen-related cell adhesion molecule) is found in EVs. In colon cancer, they are found to influence T cell functions. It also influenced T cells by activating TGF-β and SMAD signaling in colorectal cancer. T cells were inhibited by EVs produced by breast cancer cells, which delivered TGF- β directly to the immune cells, leading to cell proliferation. T cell apoptosis was induced by adenocarcinoma cells by transferring the Fas ligand, which is a type- II transmembrane protein.

Similarly, in colorectal cancer, EVs induced Fas ligand. These activities were also observed in melanoma, prostate cancer cells, and oral cancer cells, leading to apoptosis of T cells. Regarding galactin-9, EVs play a role in T cell apoptosis. In nasopharyngeal carcinoma, EVs containing galactin-9, when interacted with Tim3, promoted apoptosis of T cells. The MAPK pathway was inhibited by the miRNAs of EVs in nasopharyngeal carcinoma that reduced T cell proliferation. The metabolic checkpoint molecule arginase-1 was found in EVs generated from ovarian cancer cells, suppressing T cell responses and promoting tumor development.

#### 6.5.3. B Lymphocytes

Cancer-derived EVs’ role in B lymphocytes has also been reported recently. EVs released from esophageal cancer cell lines encouraged the development of naïve B cells into TGF-β-producing regulatory B cells, assisting immunological suppressor effects on T cell proliferation [68]. Tumor-derived EVs preferentially attach to the lymph nodes sub-capsular sinus, where a specific population of macrophages (CD169) prevents cancer EVs from spreading. During cancer, this barrier is broken, allowing tumor-derived EVs to pass via lymph nodes and activate B lymphocytes, encouraging tumor development [120].

#### 6.5.4. Macrophages

Several research studies have shown EVs produced by tumor cells to be capable of transferring cargo to macrophages. In ovarian cancer, EVs containing oncogenic miR-1246 transferred the miRNA to M2 type (anti-inflammatory) but not to M0 type (non-activated), suggesting a role of EVs in tumor progression [121]. In colon cancer, the transfer of oncogenic miR-1246 may produce IL-10 and metalloproteinase, all of which are tumor-supportive, thus leading to progression [122]. The miR-921-3p and miR-25-3p carried by EVs in liposarcoma cells promoted the secretion of IL-6, which further aided cancer progression [123]. TUC339, a lncRNA found in EVs produced by hepatocellular carcinoma cell lines, can control macrophage activation. Moreover, it promotes M2 macrophage polarization, promoting cancer cell growth. Under hypoxia, ovarian cancer cell lines produced EVs with high amounts of miR-940, causing macrophage M2 polarization [124]. In a clinical study, a glioblastoma-bearing mouse brain model, EVs extracted from glioblastoma cells carrying high levels of miRNAs (such as miR-21) were taken up by macrophages, supporting the role of EVs produced by glioblastoma cells in the TME (tumor microenvironment) [125].

#### 6.5.5. NK and Dendritic Cells

Dendritic cells are well known for activating natural killer cells, also known as NK cells [126]. They are also involved in immune evasion in the tumor microenvironment since dendritic cells can easily internalize tumor-derived EVs. When tumor-derived EVs contact T cell surface molecules, they convey signals and change their function [127]. By this, it can easily bypass host immunity against the tumor since it can interfere with antigen-presenting cells and, thus, the cytotoxic T lymphocytes may fail to recognize it [128]. Transforming growth factor-β (TGF-β) is often found on the surface of tumor-derived EVs. TGF-β is also well known for immunosuppression in patients with acute myeloid leukemia, where it was found that TGF-β is involved in suppressing NK cell function and T cell proliferation [129].

Host cells express MHC-I; to protect cells from destruction from cytotoxic T-lymphocytes (CTLs). However, MHC-I; presented by tumor-derived EVs are destroyed by EVs. The downregulation of MHC-I;/TAA complexes allows immune escape for tumors [130]. However, cells without MHC-I; are killed by NK cells [131]. Thus, to prevent this attack, tumor cells release EVs that can influence the cytotoxic activity of T cells [132]. EVs bearing NKG2D ligands act as bait for NK cells, which distracts the NK cells from attacking the tumor cells, and this helps the tumor cells to evade the immune system [133,134]. However, EVs from dendritic cells are valuable for antitumor therapy such as immunotherapy [135,136]. Further research is important to clarify how dendritic cells and NK cells can be used for antitumor therapies.

#### 6.5.6. Transfer of Mutations

Evidence shows that EVs generated from tumors contain DNA fragments that may make up the whole genome. PTPRZ1 (Protein tyrosine phosphatase receptor type Z1) –Met (PTPRZ1-Met) in EV cargo in glioblastoma cells transferred to other cells created an aggressive phenotype [137]. Anaplastic lymphoma kinase-associated mRNAs carried by EVs when transferred to susceptible cells initiated the MAPK signaling pathway. EGFRvIII (the epidermal growth factor receptor in a truncated oncogenic form)-containing EVs were picked up by recipient glioma cells that lacked this isoform, and activated MAPK and AKT pathways. In colorectal cancer, oncogenic mutant β-catenin (EV cargo) activated WNT signaling in recipient cells, leading to cancer progression. EVs in ovarian cancer also indicated SMAD 4 mutations mediated by EVs [138]. Further, a more detailed study of EVs and their relationship with mutations and their transfer mechanisms and epigenetics about tumor-derived EVs are required to obtain more knowledge in cancer biology. The tumor-derived cargoes of EVs and their role in cancer are summarized in the more detailed, still not exhaustive, Table 2.

## 7. Derived EVs and Therapy Resistance

It has been discovered that EVs induce therapy resistance to chemotherapy and targeted therapy in cancer, including multidrug resistance (MDR) [187,188]. According to the study, exosomes act as transporters of mRNAs and miRNAs, which were shown to be neoteric mechanisms in the development of chemoresistance in pancreatic cancer (PC). They were also discovered to have a role in reactive oxygen species (ROS) detoxification and gemcitabine metabolism. It is also concluded that exosome-induced PC chemoresistance is mediated by three genes: CAT, SOD2, and DCK [189].

Emerging data has indicated that EV-mediated resistance transmission occurs in acute myeloid leukemia (AML). In recent years, EVs can transfer proteins and miRNAs across AML cells, resulting in variable gene-expression patterns and cell function in receiving AML cells [190,191]. EV-mediated miRNA transfer has been shown to enhance chemoresistance in various cancers. Therefore, exosomes produced from AML patients’ BMSCs (bone marrow stromal cells) had a more distinct miRNA profile than healthy controls [191,192].

According to research, exosomes obtained from BMSCs from eight AML patients were constantly enriched in miR-155 and miR-375, compared to exosomes derived from healthy donor BMSCs [188]. A recent study also showed that pancreatic tumor cells produce exosomes indicating surface marker glypican-1, which were identified in blood samples from pancreatic cancer patients [193]. In a study, it was found that exosomes derived from pancreatic tumors enhanced liver metastasis by inducing the development of pre-metastatic regions [194]. In colorectal cancer stem cells generated from patient-derived mice xenografts or by sorting for CD133+ in colorectal cancer cell lines, EVs from cancer-associated fibroblasts promoted resistance to 5-fluorouracil and oxaliplatin inside stromal cells [195]. Another example is cisplatin resistance in head-and-neck cancer cells due to the transfer of miR-196a. It targets CDKN1B (cyclin-dependent kinase inhibitor 1B) and ING5 (inhibitor of growth family member 5), and cancer-associated adipocytes, leading to paclitaxel resistance in ovarian cancer cells due to the transfer of miR-21, which binds to the APAF1 (apoptotic protease activating factor 1) coding sequence and suppresses APAF1 expression [196,197]. Intriguingly, both in vitro and in vivo, miR-21-carrying EVs from neuroblastoma cells promoted the production of monocyte EVs expressing miR-155. This enhanced cisplatin resistance in neuroblastoma cells by entering these cells and inhibiting TERF1 (telomeric repeat binding factor 1) [198]. EVs from drug-resistant cancer cells and tumor microenvironment cells are randomly implicated in transferring anti-cancer drug resistance. This is also evidenced by several previous research groups across solid and not solid cancer types, adding to anti-cancer therapy problems [199].

### 7.1. EVs and Multi-Drug Resistance (MDR)

**Figure 2:**
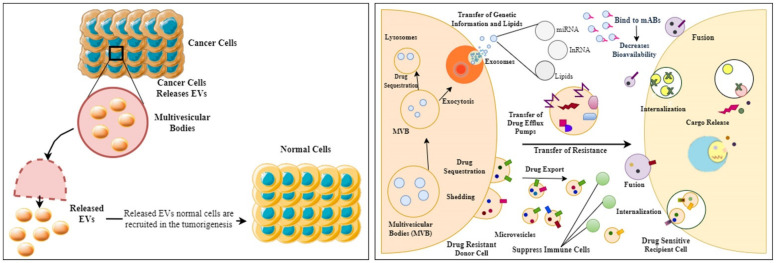
Diagrammatic representation showing how cancer cell-derived EVs promote tumorigenesis in normal cells and later help the drug-sensitive cells to transform into drug-resistant cells, thus owing to failed chemotherapy.

MDR is a characteristic of cells that are resistant to a variety of structurally and functionally diverse drugs. Resistance can be complex and result from various processes, such as impairing drug efficacy, including reduced drug absorption, altered drug metabolism, drug target mutation or expression modification, cell death suppression, increased DNA repair, and so on [200,201]. MDR can be caused by various factors, including ATP-binding cassette (ABC) transporters that pump out chemotherapeutics and oncogene mutations that make cancer cells resistant to previous treatments, such as emerging modification of cancer cells to the microenvironment, surviving cancer stem cells (CSCs) that evade conventional therapies, activated cell growth variables, and cell defense systems, among others [202]. Researchers have also discovered that EVs are linked to the transmission of anti-cancer drug resistance from cell to cell in various malignancies, based on pre-clinical and clinical specimen investigations [199]. Furthermore, intracellular integration of EVs generated by drug-resistant cancer cells can cause drug-sensitive cancer cells to become drug-resistant [200]. MDR has become a serious issue in cancer treatment, and it is one of the leading causes of chemotherapy failure. Therefore, multiple resistance mechanisms are common in MDR cancer cells [203]. According to recent studies, altered metabolic pathways aid cancer cells in proliferating, adapting their metabolism to nutrient-depleted circumstances, and developing drug-resistant phenotypes [204]. In a study, researchers have shown that EVs produced by MDR cells can cause drug-sensitive cancer cells to undergo a metabolic change (leading to an MDR phenotype) [200]. Compared to the EVs shed by drug-sensitive counterpart cells, MDR cells release a distinct population of EVs (more microvesicle-like EVs and fewer exosomes) [205]. The primary drug transporter P-glycoprotein (P-gp) expression has been seen in the generality of cancers with MDR phenotype. It is associated with tolerance to at least 20 different chemotherapeutic drugs encoded by the multidrug resistance protein 1 gene (MDR1 or ABCB1) [206]. Wang et al. demonstrated that chemotherapeutic drugs cause EVs secretion and recycling by dysregulating Rab8B and Rab5, resulting in a notable increase in ABCB1 intercellular transfer, and showed that Rab8B plays a role in MDR development [207]. P-gp could be transported from drug-resistant to drug-sensitive cancer cells by migrating EVs, increasing acquired therapy resistance both in vitro and in vivo, according to a substantial number of studies [208,209]. Cancer cell-derived EVs, and how they influence drug-sensitive cells to drug resistance, is presented in Figure 2**.**

Breast cancer was first investigated concerning drug resistance that is linked to EV structures, and the role of EVs in mediating drug resistance was reported by Chen et al. [210]. Jaiswal et al. also revealed that doxorubicin-resistant breast cancer cells secrete large amounts of secretory miRNAs, imparting drug-resistant phenotypes to recipient cells [211]. MCF-7/Adr and MCF-7/Doc, respectively, contain EVs from adriamycin- and docetaxel-resistant cell lines, where confocal microscopy and flow cytometry were used to track the absorption of fluorescently (PKH26) labeled EVs by MCF-7 cells, which conferred resistance to previously drug-sensitive MCF-7 cells. Following the transfer of EVs from the docetaxel-resistant variations, levels of miR-100, miR-222, miR-30a, and miR-17 were found to be considerably elevated in formerly drug-sensitive MCF-7 cells [84]. Other cancers, such as prostate and lung cancer, have shown drug resistance concerning EVs. Therefore, researchers conducted the first studies on prostate cancer and EVs with drug resistance, focusing on docetaxel and examining 22Rv1 and DU145 cell lines, as well as their corresponding docetaxel-resistant variants 22Rv1RD and DU145RD [199].

According to some research, drug-resistant cells generate more EVs than drug-sensitive cells [212]. According to research, EVs secreted from an anaplastic lymphoma kinase (ALK)-tyrosine kinase inhibitor (TKI)-resistant subclone of ALK-translocated lung adenocarcinoma cell lines can cause drug resistance in sensitive subclones [213]. Furthermore, several studies have found a direct correlation between certain drug-resistance mediators and molecules involved in EVs formation, corroborating the results that drug-resistant cells produce more EVs than sensitive cells [7]. Annexin A3, which is implicated in ovarian cancer cell resistance to platinum and has been found in EVs from a similar kind of cancer resistant to cisplatin, seems to be responsible for an upsurge in EVs generation in the same cells [214]. In the presence of HER2-carrying EVs, trastuzumab was also ineffective in inhibiting breast cancer cell growth. When this receptor (which dimerizes with EGFR or HER3) is activated, it promotes the creation of EVs [215]. An elevation in miR-155 expression rates in cells transfected with pre-miR-155 produced an increase in the emission of EVs and a rise in miR-155 expression levels on the released EVs’ content, according to thorough research in pancreatic ductal adenocarcinoma cell lines. These EVs transported miR-155 to additional pancreatic ductal adenocarcinoma cancer cells, causing recipient cells to develop gemcitabine resistance [216]. The list of proteins/enzymes from EVs and their role in cancer drug resistance is represented in Table 3.

### 7.2. Drug Efflux Pumps and Their Transfer

The potency of cytotoxic drugs is dependent on their ability to accumulate intracellularly in cancer cells. Hence, increased drug efflux is a key factor of chemoresistance. In the context of drug resistance, the existence of drug efflux pumps in EVs is significant, not just because the active sequestration of drugs in EVs may be mediated by both P-gp and ABC (ATP-binding cassette) transporter G2. EVs that transport the drug-resistance from drug-resistant to drug-sensitive cells facilitate the in vitro and in vivo promotion of acquired therapy resistance [79,233]. When compared to their drug-resistant cells, the orientation of these transporters in some EVs may be inverted, potentially favoring drug influx rather than efflux into EVs. The existence of selective P-gp/MDR-1 mRNA in EVs produced from doxorubicin-resistant osteosarcoma cells demonstrates that drug-resistant tumor cells use various methods to spread resistance to sensitive cells. Furthermore, MDR proteins are transferred directly to sensitive cells, or the mRNA that encodes them is transferred, leading to various drug-resistance processes [234].

Many studies have demonstrated the non-genetic acquisition of P-gp, causing drug resistance or enrichment on this drug efflux pump in EVs released by drug-resistant cells. These include castration-resistant prostate cancer cells and docetaxel, doxorubicin, and adriamycin-resistant breast cancer cells [203]. For the first time, this “non-genetic acquisition” of P-gp in the setting of MDR was demonstrated in a neuroblastoma model, wherein a drug-sensitive cell line obtained functional P-gp subsequent co-culture with a drug-resistant equivalent cell line [208]. Moreover, exosomal P-gp transport provided taxane resistance to prostate and ovarian cancer cells. In contrast, osteosarcoma cells were found to disseminate their capacity to escape doxorubicin treatment by the EV-related transfer of MDR-1 mRNA [203]. P-gp is enclosed in the vesicular membrane and transported to recipient cells (sensitive drug cells), exposing it to their surface [206]. It is uncertain if the EV cargo is transferred selectively or randomly across cells. Hence, P-gp was transferred to breast and lung cancer recipient cells via EVs produced by drug-resistant leukemia cells with no cell-type selectivity [82]. Furthermore, this cargo was found to be transmitted (together with MRP1 or multidrug-resistance-associated protein 1) to both malignant and non-malignant sensitive cells.

In contrast, P-gp was exclusively delivered to malignant cells by EVs from breast cancer cells in vivo [235]. Other drug efflux exporters, such as ABCG2 or ABCA3 (ATP-binding cassette transporter A3), have been horizontally transmitted by EVs, promoting cytotoxic agent excretion and modifying chemoresistance in cancer cells [206]. Thus, these MDR-carrying EVs might be transported to other cells inside the heterogeneous tumor or stromal cells within the tumor microenvironment, affecting their treatment response.

### 7.3. Anti-Apoptotic Pathways and Their Development

Apoptosis regulates cell populations during ontogeny and in adult tissues. It is a well-known process for its role in controlling tissue turnover and homeostasis, in which the increase of cell populations is countered by regulated cell death. Carl Vogt, a German scientist, first characterized the apoptotic mechanism in 1842, and the name “apoptosis” was coined by the John Foxton Ross Kerr study in 1972 [236]. Horizontal transmission of particular bioactive cargo by EVs can potentially affect cell cycle control and apoptotic processes in recipient cells. Suppression of apoptosis’ tumor-suppressor role resulted in classifying a distinct type of oncogenes, with BCL2 (B cell lymphoma 2) as the prototypic member, that might enhance cell survival by inhibiting apoptosis and inflicting an oncogenic imbalance on the cell birth/cell death equation [237].

Additionally, several cancer types have mutations in the apoptosis effector protease caspase-8, and the survival pathway PI3K/Akt/mTOR is commonly downregulating in malignancies [238]. EVs can elicit pro-survival and anti-apoptotic signals in tumor cells, leading to resistance to a wide spectrum of chemotherapeutics. The PI3K/AKT pathway is one of the main oncogenic cascades implicated in cancer cell proliferation [239]. Other research has reported EV-mediated drug resistance by targeting apoptosis regulators and encouraging anti-apoptotic pathways in recipient cells, such as the Bcl-2/BAX signaling [240]. In EVs generated from chronic myeloid leukemia cells, mRNA encoding inhibitors of apoptosis proteins (IAPs) were discovered [82]. P-gp and microRNAs (miR-27a, miR-451, and miR-21) linked to P-gp expression were accumulated in recipient cells via EVs from drug-resistant chronic myeloid leukemia cells. The inhibitors of apoptotic proteins XIAP, IAP, and a survivin member of the IAP family of proteins has also been found in EVs obtained from various tumor types, including cervical and prostate cancer, and they improve defense against genotoxic stress and proton irradiation [206]. These EVs caused drug resistance in drug-sensitive recipient breast and lung tumor cells, implying that drug resistance and apoptosis escape are multiple processes.

### 7.4. Transfer of Proteins and Lipids

In a recent paper on proteasome variations in EVs obtained from two cisplatin-resistant and one sensitive oral squamous cell carcinoma cell lines, researchers revealed 77 differentially expressed proteins in EVs from both resistant cell lines, most of which were dysfunctional and involved in EGFR-associated channels [241]. Multiple EV biogenesis and function elements have been correlated to lipids, and lipids have also been associated with EV-mediated drug resistance. Many cancers, notably prostate cancer, have enhanced absorption and de novo production of cholesterols and cholesteryl esters and the build-up of triacylglycerol as energy reservoirs. Although EVs are known to transport lipids and lipid-related proteins, they can influence the function of target cells [242]. Ceramide, which is involved in EVs’ biogenesis and cargo loading, may potentially have a role in drug resistance by P-gp [243]. Trajkovic et al. also reported an exosome biogenesis route that is not dependent on ESCRT (endosomal sorting complex required for transport). It is mediated by sphingolipid ceramide [244].

Moreover, a lipidomic analysis of EVs recovered from a gefitinib-resistant lung cancer cell line, its sensitive parent cell line, found variations in the expression of 35 phospholipids. After subjection to melphalan and bortezomib, the expression of acid sphingomyelinase from multiple myeloma cell lines was elevated in EVs, which results in transferring a drug-resistant phenotype to chemo-sensitive cells [245].

## 8. EVs as a Diagnostic Tool

Developing accurate biomarkers for early cancer detection is essential to minimize patient mortality. The ideal diagnostic approach would use non-invasive methods to detect tumor-specific biomarkers during pre-metastatic stages. Body fluid samples, including circulating tumor cells, tumor DNA, tumor RNA, and EVs, may indeed be unique sources and excellent biomarkers for cancer diagnosis, in contrast to standard approaches using biopsies. According to studies, EVs are found in all body fluids and are a significant source of biomarkers that can identify the state of any pathological disease, including cancer [246]. Circulating EV miRNAs unique to tumors can be employed as diagnostic and prognostic biomarkers to detect hepatocellular carcinoma, breast cancer, lung cancer, gastrointestinal cancer, colon cancer, pancreatic cancer, melanoma, ovarian cancer, and prostate cancer. According to Taylor and colleagues, serum biomarkers such as miRNA214, miRNA-205, miRNA-203, miRNA-141, miRNA-200-a, -b, -c, and miRNA-21 are also abundant in sera-derived exosomes of ovarian tumor patients [247]. PDL1 in plasma-circulating EVs is considerably higher in patients with metastatic melanoma. PDL1 levels on the surface of circulating EVs correlate with disease progression in melanoma patients, and the measurement of PDL1 levels in plasma samples allows patients to be stratified based on disease progression [76]. Glypican-1 (GPC1) levels in the blood have also been proposed as a possible prognostic and diagnostic biomarker for pancreatic cancer, with EV GPC1 levels being assessed in the blood [248]. When comparing prostate cancer patients’ urine exosomes to controls, transcriptome profiling revealed increased levels of PCA-3 (prostate cancer antigen 3 gene) and TMPRSS2-ERG (transmembrane protease serine 2-ETS-related gene). However, circulating vesicle transcriptome analysis may indicate prostate cancer malignancy status, due to the diversity of EVs. There are no common markers that can be applied to all EVs.

### Biomarkers

**Figure 3:**
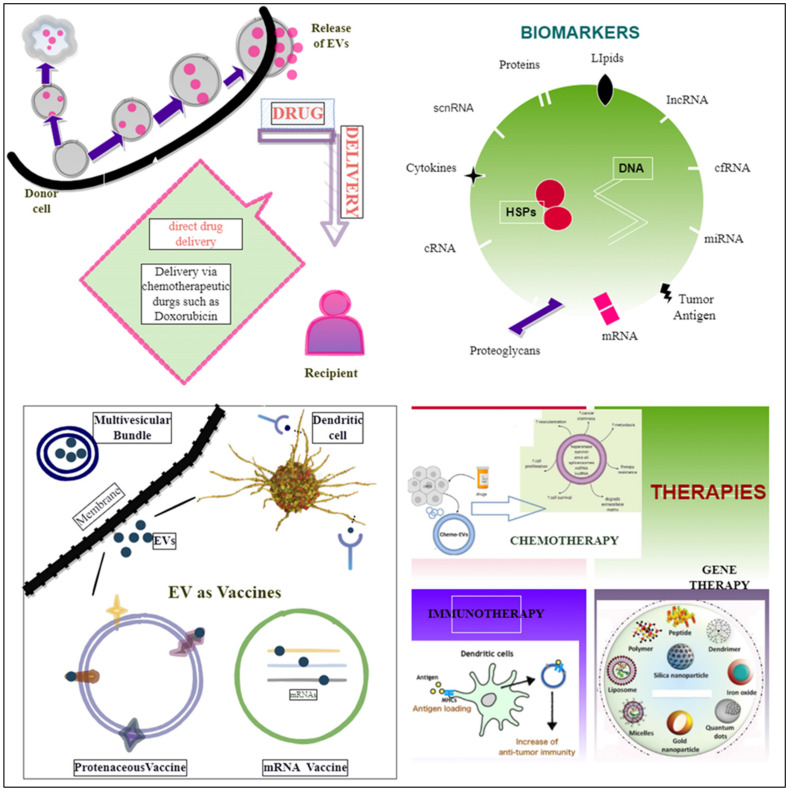
Diagrammatic representation revealing EVs’ role as biomarkers, vaccines, and in different therapies.

EVs are becoming more widely recognized as potential diagnostic or prognostic biomarker sources as they provide non-invasive, near-constant access to circulating information on the tumor’s condition [249]. In a study of tumor-derived circulating EVs from patients who had a poor response to chemotherapy, the breast cancer resistance protein (BRCP) was shown to be increased at the mRNA and protein levels. MUC1 (known as mucin 1, cell-surface-associated) was also shown to be co-expressed with BCRP, suggesting that it might be used as a marker of chemotherapy response [250]. Molecular miRNAs may be actively released into exosomes and microvesicles. Molecular miRNAs can be taken up into secondary cells as per the circulating (extracellular) biomarkers [251]. The use of EV-miR-21 was also investigated as a biomarker in oral squamous cell carcinoma [252]. Castration-resistant prostate cancer responses to treatment are predicted by miR-34a, an intracellular and exosomal biomarker [251]. MiR-200c-3p, miR-21–5p, and miR-28–5p were decreased with better immunotherapy responses in lung cancer [253]. Protein biomarkers, such as CA125 and prostate-specific antigens (PSAs) for ovarian and prostate malignancies, were the first targets utilized in cancer liquid biopsy [254]. The Food and Drug Administration (FDA) has authorized ExoDx™ Prostate IntelliScore, the first exosome-based liquid biopsy test, which analyses exosomal RNA for three distinct biomarkers on urine specimens: PCA3, TMPRSS2: ERG, and SPDEF (SAM-pointed domain-containing ETS transcription factor) [255]. SERPINA1 (alpha-1 antitrypsin) and H2B1K (histone H2B type 1-K) were discovered as promising bladder cancer biomarkers for prognosis using urine EVs and proteomic data from diseased patients versus healthy persons [256]. Other than microRNAs, multiple circulating myeloma EV cargoes have been identified as potential disease biomarkers. Liquid biopsy is a procedure that involves detecting and analyzing biological markers in a biofluid sample such as blood, urine, cerebrospinal fluid, and seminal plasma to assess disease and determine therapy choices [257]. It becomes easy to measure the heterogeneity of the entire tumor and has overcome the barrier of fast diagnosis via liquid biopsy [258]. The biofluid samples from certain cancer patients, such as prostate, lung, and ovarian cancer patients, have found high levels of EVs, emphasizing, therefore, the diagnostic importance of EVs [81], [259,260,261]. Bioactive molecules on the EV surface (adhesion molecules, proteoglycans, lipids, and tumor antigens), as well as molecular content makes up the EV cargo (DNA, mRNA, microRNA, LncRNA, circular RNA, metabolites, heat shock proteins, enzymes), as shown in Figure 3, and the presence of these functional properties enhances the overall therapeutic and diagnostic potential of EVs in cancer therapy [257,262].

Recently, research published by Hoshino et al. used extracellular vesicles and particles (EVP) to distinguish normal and tumor tissues using machine-learning classification of plasma-derived EVP cargo, which showed 90% specificity and 95% sensitivity in detecting cancer; thus, they proved the usefulness of the EVP markers [263]. Further research is required in such upcoming techniques to further explore the possible use of EVs as biomarkers.

## 9. EVs as a Tool for Cancer Therapy

Even though cancer is one of the most researched diseases in humans, it has an unclear pathophysiology, owing to the quite-heterogeneous nature of the disease [264]. EVs, especially exosomes, provide fresh light into cancer biology and can be used for diagnostic and therapeutic purposes. EVs impact tumor development, metastasis, and treatment effectiveness, leading to drug resistance due to cell-to-cell communication. They maintain their stability at high temperatures and are safeguarded from deterioration through their lipid bilayer. These characteristics make them suitable biomarkers and drug-delivery carriers [265]. Below, we have discussed, in detail, the potential of EVs as a therapeutic tool to deliver drugs for and treat cancer.

### 9.1. Drug Delivery Vehicle

The above-cited properties of EVs, such as their small size, the presence of adhesive compounds on their surface, and their role as biomolecule carriers, make them ideal for drug delivery [266]. Direct drug loading involves loading drugs directly into an EV without going through EV-derived cells. This technique ensures drug loading accuracy while reducing other conflicting compounds to a minimum. Conventional chemotherapy drugs have inadequate targeting and adverse effects. Because of natural exosomes, these EVs can improve the targeting of chemotherapy drugs. PTX (paclitaxel) is currently the most extensively explored exosomal chemotherapeutic drug. Although PTX has a low water solubility in clinical applications, it is frequently dissolved in Cremophor EL and ethanol to increase solubility. Cremophor EL, on the other hand, can produce hypersensitivity responses, such as hyperlipidemia and red blood cell aggregation, and permanent sensory neuropathy in the long run [267]. In a study, researchers encapsulated PTX in milk-derived exosomes and produced continuous PTX release for up to 48 h while maintaining optimal drug stability. PTX was encapsulated in exosomes and given orally. The systemic and immunogenic responses were dramatically reduced compared to straight intravenous treatment [268].

EVs have been gaining prominence as new drug-delivery vehicles due to their structural resemblance to liposomes [269]. Liposomes have demonstrated their efficacy as a new drug carrier and have been actively used for drug delivery since their composition is remarkably similar to the cell membrane [270]. Liposomal research has set the groundwork for the further investigation of their physicochemical characteristics. Although EVs are naturally formed by cells and can readily transmit the required drugs, they are a superior alternative and more efficient than liposomes [271]. Exosomes, identical to liposomes, have a lower loading efficacy (5–50% depending on methodologies) than liposomes with higher efficacy (30–90% depending on methods). The use of liposome- and exosome-EVs as hybrid nano-carriers in improved drug-delivery systems has recently been proposed. Sato YT et al., for example, used the freeze–thaw approach to fuse the membranes of exosomes and liposomes, resulting in a hybrid exosome. Uptake assays demonstrated that changing the membrane lipid makeup, or features of foreign lipids in hybrid exosomes, can modify the associations between exosomes and recipient cells [272].

RNA therapies have much promise since they can alter gene expression; nevertheless, the large polar RNA molecule cannot cross the cell membrane and is soon digested by extracellular RNases [273]. Enveloping therapeutic RNA into synthetic nanoparticles or conjugating RNA to particular ligands targeted to increase absorption are two current standard techniques for overcoming these limitations. In several investigations, EVs are an excellent tool for delivering small interfering RNAs and other synthetic molecules for therapeutic reasons. In a recent study, electroporation was used to create significant amounts of MSC-derived EVs loaded with an anti-KRASG12D siRNA, enhancing survival in a mouse model of pancreatic cancer. However, there is some question about the effectiveness of electroporation for loading siRNA into EVs [274]. A study revealed that sonication might deliver oncogenic siRNAs to EVs with high efficiency. EVs were loaded with small RNAs without aggregation, and these therapeutic EVs were carried up by HEK293T and MCF-7, culminating in HER2 knockdown and tumor growth suppression [275].

### 9.2. EVs as Vaccines

EVs as biological carriers in cancer immunotherapy have recently been explored. A novel cancer therapy method is using EVs to carry compounds capable of eliciting an immune response to kill cancer cells. EVs have been investigated for novel cancer vaccines, either generated from APCs or obtained directly from tumors [276]. Cancer and immune cell-derived EVs can stimulate recipient cells’ immune systems. Because of various stimulatory molecules on their surface, including heat-shock proteins and several tumor antigens, these cancer-derived EVs are thought to be potential pro-immune elements. There is a multitude of evidence supporting EVs as strong immunosuppressive agents [277].

Many studies have found dendritic cell-derived exosomes (DEX) to modulate immunological responses to cancer. DEXs stabilize vesicles and are difficult to degrade or inactivate. DEXs are high in membrane proteins such as major histocompatibility complex class I (MHC class I), MHC class II molecules, CD63, CD81, and integrin, according to research, and have a substantial immune activation impact [278]. In in vivo conditions, the application of EVs as tumor vaccines has demonstrated promising anticancer benefits, and clinical trials on EVs have yielded promising outcomes. A novel DEX vaccine containing antigens and matured with either the TLR-3 (toll-like receptor-3) ligand-induced robust activation of melanoma-specific CD8+ T cells, as well as the selection of cytotoxic CD8+ T cells, NK, and NK-T cells to the tumor site, resulting in noticeably decreased tumor growth and improved survival rates [279]. To produce ovalbumin-containing EV antigens in vivo, plasmid DNA vaccines expressing EV-associated antigens were later employed as vaccines in mice, either exposed on the surface of vesicles or integrated into membrane-enclosed virus-like particles [280]. The number of updates on EVs produced from tumors or immune cells to induce antitumor responses and cancer suppression is growing, apart from the fact that several studies support tumor EV-driven immune suppression [281].

### 9.3. EVs in Gene Therapy

Exosomes containing the gene or drug of interest have been isolated from donor cells. The endosomal pathway, hijacked by viruses and utilized for improved RNA delivery in vivo, is where exosomes are produced from engineered cells [282]. Many researchers have spontaneously used the genetic material conveyed by foreign bodies, and many cancer-related studies have used microRNAs to explore exosomes. Exosome-derived microRNAs have been shown to stimulate cell motility, inflammation, immunological response, angiogenesis, invasion, pre-metastatic niche creation, and metastasis by target-gene transcriptional inhibition [279].

By introducing DNA fragments, miRNA, siRNA, and lncRNA in target cells via viral vectors such as human immunodeficiency virus (HIV), adeno-associated viruses, or naked plasmid DNA, gene therapy can reverse aberrant gene expression, induce suicide gene expression, modulate the immune response, and inhibit angiogenesis [283]. In a study, HEK-293T cells engineered to express the suicide gene and a protein-cytosine deaminase (CD) in high levels amalgamated to uracil phosphoribosyl transferase (UPRT). The CD-UPRT mRNA/protein composite loaded into EVs displayed tumor-cell-killing activity, indicating that suicide gene-carrying EVs are significantly vital in cancer treatment [284,285]. Dendritic cell-derived EVs containing miR-155 and miR-146a may be successfully transferred across immune cells in vivo and influence the inflammatory response in mammals positively and negatively, respectively [286]. Moreover, the most intriguing research is that of Usman et al., who demonstrated that inducing antisense miR-125 lowers the development of acute myeloid leukemia (AML) in vivo, in addition to proving the benefits of employing EVs derived from blood cells for gene therapy. This was accomplished by injecting antisense oligonucleotide into 3.3 × 1012 EVs, which was then injected into tumor-bearing mice [287]. In recent research, engineered EVs delivering specific RNA oligonucleotides against Che-1/AATF polymerase were used to limit the possibility of precursor B cell acute lymphoblastic leukemia [288]. The targeted delivery of DNA plasmid vectors (e.g., the CRISPR/Cas9 system) to recipient cells is a promising application for therapeutic genome editing. Hybrid EVs with CRISPR/Cas9 expression vectors, for example, successfully manipulate genes in mesenchymal stem cells (MSCs) in vivo [289]. In another study, cancer cell-derived EVs were employed to efficaciously distribute CRISPR/Cas9 plasmids for the targeted suppression of poly (ADP-ribose) polymerase-1 (PARP-1), which promotes cell death (apoptosis) in ovarian cancer cells, improving their cisplatin sensitivity [290]. The role of EVs as biomarkers, in gene therapy and as vaccines, is represented in Figure 3.

#### Future Prospects of EVs in Gene Therapy

Gene therapy with EVs is a research topic that is the focus of many companies and researchers worldwide [291,292]. EV mediated gene therapy requires EV engineering, where different strategies are used to achieve the desired outcomes [293]. Codiak BioSciences, a biopharmaceutical company, recently reported working on engineered EVs [294]. It developed ExoIL-12, which is an engineered exosome with a scaffold protein that displays functional interleukin-12 (IL-12) on their surfaces. IL-12 is a proinflammatory cytokine that can stimulate the immune system to attack cancer; however, its results for clinical trials are awaited [294,295]. Another engineered EV, named exoSTING, was developed by the same company. It has a STING (stimulator of interferon genes) agonist loaded internally, which expresses exosomal protein PTGFRN to facilitate uptake in specific tumor resident antigen-presenting cells, or APCs, to generate a local immune response. However, it has yet to enter phase 3 trials [296,297]. Another engineered exosome is exoASO-STAT6 that uses the same scaffold but to, instead, attach an antisense oligonucleotide, which is designed to silence STAT6, a transcription factor for tumor-associated macrophages that promotes cancer progression [298]. Another company, named OmniSpirant THERAPEUTICS, is developing inhaled engineered EVs based on an inhaled exosome technology platform. As we know, mutant CTFR genes cause cystic fibrosis. The company is developing engineered stem cell exosomes that carry the working copy of the CTFR gene, which are carried by the inhaled aerosol to the damaged lung cells. Exosomes deliver the working copy of the CTFR gene and help to regenerate the lung tissues [299,300]. Carmine Therapeutics, an EVX Ventures company based in Cambridge, is currently working with EVs of red blood cells (RBCEVs). In an experiment, they used antisense oligonucleotide (ASO) delivered by RBCEVs to treat leukemia. 125b-ASO-loaded RBCEV appeared to inhibit the uptake of miR-125b, which is an oncogenic miRNA for leukemia, prostate cancer, and breast cancer. Inhibiting the uptake of miR-125 by the cancer cells resulted in reduced proliferation. Another experiment showed that a gRNA designed by the same team of Carmine Therapeutics targeted miR-125b-2 locus with a mutation site in the seed sequence of the miRNA. After a 2-day treatment of mice with RBCEVs loaded with Cas9 mRNA and 125b-gRNA, the expression of miR-125b and miR-125a was reduced by 98 percent and 90 percent in MOLM13 cells, respectively [287]. However, there are yet many challenges with EV-based gene therapy; these may include delivery, targeted treatment, etc. [298]. Thus, further research with existing techniques or innovating new techniques is required to understand and study the possible use of EVs for gene therapy to treat cancer soon.

## 10. Conclusions

Various studies in recent years indicate the role of EVs in aiding the tumor microenvironment. These involve promoting cell proliferation, escaping cell apoptosis, sustaining angiogenesis, promoting metastasis, etc. It has also been observed that tumor-derived EVs aid in evading the immune response. Cancer cell-derived EVs transform normal cells into tumorigenic cells at the epigenetic level because they release cargoes containing DNA and RNA from the affected cells to the normal cells due to their transformation. Several studies reveal that the cargoes released from the tumor-derived EVs affect the cell’s metabolism by altering the pathways to ones that can easily support the tumor microenvironment during different hallmarks of cancer; for example, the activation of the MAP/ERK pathway leads to the initiation of the tumor. Other studies also found that EVs associated with TGF-β aid the tumor microenvironment in a variety of ways, including the development of immunosuppressive cell variants, aiding metastasis, and so on. Therefore, the affected EVs carry cargoes such as miRNA, mRNA, lncRNA, DNA, proteins, lipids, etc., released from tumor cells to the normal cells resulting in the normal cell’s transformation and aiding the tumor microenvironment [7].

Decades back, the role of EVs in providing resistance to cancer treatment was unknown. However, recently, many studies are being conducted, which confirms that EVs released from tumor cells can greatly impact therapies, mostly chemotherapy, as EVs aid cells to develop drug efflux mechanisms. Thus, it prevents the essential drug from moving inside the cell. EVs also contribute to transforming a drug-sensitive cell into a resistant one. Recent studies also suggest the development of multiple drug-resistant cells caused by EVs; this poses a major challenge, because cancer cells become resistant to major anticancer drugs, invariably leading to chemotherapy failure [200].

On the other hand, EVs are useful since they are a good biomarker for detecting various kinds of cancer. EVs associated with tumor cells often tend to show some differences from normal cells, which may help identify or detect cancer cells. In contrast, EVs are also useful in cancer therapy since cells that have absorbed drugs tend to release EVs that work as a drug-delivery vehicle and tend to stop cancer [9,266]. Not only this, but EVs are also useful since they can be used for gene therapy and also as a vaccine [276]. Thus, EVs are both useful as well as harmful, depending on the nature of the source of the EVs from where they are released [9,301]. Although there has been much research about EVs and their possible use, there are also clinical limitations ranging from the isolation, quantification, dosing, possible uptake assessments, and inappropriate controls, which are all the fields that need to be further addressed. This can be accomplished through existing techniques or new techniques and strategies, which need to be put in place [302,303]. Therefore, more studies are needed to clarify the role of tumor-derived EVs in different hallmarks of cancer; in contrast, studies are also needed regarding to how the EVs can be positively utilized as biomarkers and to help stop cancer progression.

## Figures and Tables

**Table 1:** List of source and functions of EVs (cargoes) released from normal cells.

S. No.	Source of EVs	Functions of EVs/EV Cargoes	References
1	CD9-containing vesicles	Sperm and egg fusion	[35]
2	Oocyte	Remove sperm receptor to prevent polyspermy	[36]
3	Embryonic stem cells	EVs to communicate with environment in blastocyst; trophoblast migration and invasive properties	[37]
4	B Lymphocytes	To activate T cells	[38]
5	Antigen-presenting cells	RNA cargo influencing immune cell behavior	[39]
6	Dendritic cells	miRNA cargo represses mRNA expression target in acceptor dendritic cells	[39]
7	Regulatory T cells	miRNA cargo suppresses inflammatory responses of helper T1 cells	[40]
8	Neurons and glia cells	Facilitate intercellular communication	[41]
9	Neurons	mi-124a cargo uptake by astrocytes upregulates expression of GLT1	[42]
10	Oligodendrocytes	Neurons intake myelin proteins and oxidative stress-protective proteins, causing changes in neuronal firing rates and gene-expression profiles	[43]
11	Oligodendrocyte	Inhibiting differentiation and myelin formation	[44]
12	Schwann cells	Enhance regeneration capacity after sciatic nerve injury	[45]
13	Microglia	Suppress the production of sphingolipid ceramides and sphingosine to regulate neuronal excitability	[46]
14	Cells of human blood from wound site	EVs expose a highly procoagulant tissue factor, implying that EVs play a role in hemostasis	[47]
15	Endothelial cells	Matrix metalloproteinases cargoes enhance matrix degradation and promotes angiogenesis	[48]
16	Platelets	Promotes cell proliferation, the migration of endothelial cells, and vessel formation	[49]
17	Lymphocyte-derived EVs	Prohibits VEGF pathway and, thus, suppresses angiogenesis and augments oxidative stress	[50]
18	Stem-cell-derived EVs	Bioactive cargoes have regenerative abilities	[51]

**Table 2:** List of cancer types and released EVs cargo and their role in cancer hallmarks.

S. No.	Cancer Type	EV Cargo Type	Role/Cancer Hallmarks	References
1	Acute Leukemia	miR-116	Cell proliferation and escape from apoptosis	[68]
2	Acute Leukemia	miR-118	Cell proliferation and escape from apoptosis	[68]
3	Brain cancer	miR-181c	Cell invasion and metastasis	[105]
4	Breast cancer	miR-1246	Cell proliferation and escape from apoptosis	[139]
5	Breast cancer	miR-210	Sustaining of angiogenesis	[111]
6	Breast cancer	miR-200 family	Cell invasion and metastasis (also highly involved in epithelial to mesenchymal transition)	[140]
7	Breast cancer	miR- 205	Cell invasion and metastasis (also highly involved in epithelial to mesenchymal transition)	[140]
8	Breast cancer	Caveolin-1	Cell invasion and metastasis	[141]
9	Breast cancer	miR-122	Reprogramming energy metabolism	[142]
10	Breast cancer	GSTP1	Reprogramming energy metabolism	[78]
11	Breast cancer	miR-122	Reprogramming energy metabolism	[78]
12	Cholangiocarcinoma	miR-205	Cell proliferation and escape from apoptosis	[143]
13	Cholangiocarcinoma	miR-205-5p	Cell invasion	[143]
14	Colon cancer	DeltaNp73	Cell proliferation and escape from apoptosis	[143]
15	Colon cancer	miR-193a	Cell proliferation and escape from apoptosis	[144]
16	Colon cancer	miR-200b	Cell proliferation and escape from apoptosis	[145]
17	Colon cancer	miR-25-3p	Sustaining of angiogenesis	[146]
18	Colon cancer	Wnt5b	Cell invasion and metastasis	[147]
19	Colon cancer	AREG	Cell invasion and metastasis	[148]
20	Colon cancer	β-catenin (mutant)	Transfer mutations	[149]
21	Colon cancer	miR-1246	Evade immune response and promote inflammation	[122]
22	Colon cancer	Carcinoembryonic antigen related cell adhesion molecule	Evade immune response and promote inflammation	[150]
23	Colorectal cancer	miR-10b	Evade immune response and promote inflammation	[151]
24	Colorectal cancer	lnRNA PVT1	Cell proliferation and escape from apoptosis	[152]
25	Colorectal cancer	miR200b	Cell proliferation and escape from apoptosis	[152]
26	Esophageal cancer	miR-93-5p	Cell proliferation and escape from apoptosis	[69]
27	Esophageal cancer	miR-21	Cell proliferation and escape from apoptosis	[70]
28	Esophageal cancer	lncZEB1-AS1	Cell proliferation and escape from apoptosis	[153]
29	Gastric cancer	lncRNAZFAS1	Cell proliferation and escape from apoptosis	[75]
30	Gastric cancer	miR-423-5p	Cell invasion and metastasis	[154]
31	Gastric cancer	miR-27a	Evade immune response and promote inflammation	[155]
32	Glioblastoma	RBM11 (splicing factor)	Cell proliferation and escape from apoptosis	[99]
33	Glioblastoma	CLIC1	Cell proliferation and escape from apoptosis	[156]
34	Glioblastoma	lncRNA CCAT2	Sustaining of angiogenesis	[157]
35	Glioblastoma	lncRNA POU3F3	Sustaining of angiogenesis	[158]
36	Glioblastoma	miR-21 (VEGF- upregulate expression)	Sustaining of angiogenesis	[159]
37	Glioblastoma	CXCR4	Sustaining of angiogenesis	[160]
38	Glioblastoma	miR-148a	Cell invasion and metastasis	[161]
39	Glioblastoma	PTPRZ1-MET (fusion gene)	Transfer mutations	[137]
40	Glioblastoma	EGFRvIII (oncogenic receptor)	Transfer mutations	[162]
41	Glioblastoma	miR-210	Evade immune response and promote inflammation	[125]
42	Glioblastoma	PD-L1	Evade immune response and promote inflammation	[163]
43	Head-and-neck squamous cell carcinoma	EPHB2	Sustaining of angiogenesis	[164]
44	Hepatocellular carcinoma	lncRNA TUC339	Cell proliferation and escape from apoptosis	[164]
45	Hepatocellular carcinoma	CXCR4	Cell proliferation and escape from apoptosis	[164]
46	Hepatocellular carcinoma	SMAD3	Cell proliferation and escape from apoptosis	[165]
47	Hepatocellular carcinoma	miR-93	Cell proliferation and escape from apoptosis	[166]
48	Hepatocellular carcinoma	miR-103	Cell proliferation and escape from apoptosis	[167]
49	Hepatocellular carcinoma	miR-584	Cell proliferation and escape from apoptosis	[168]
50	Hepatocellular carcinoma	miR-517c	Cell proliferation and escape from apoptosis	[168]
51	Hepatocellular carcinoma	miR-378	Cell proliferation and escape from apoptosis	[168]
52	Hepatocellular carcinoma	miR-520f	Cell proliferation and escape from apoptosis	[168]
53	Hepatocellular carcinoma	miR-142-5p	Cell proliferation and escape from apoptosis	[168]
54	Hepatocellular carcinoma	miR-451	Cell proliferation and escape from apoptosis	[168]
55	Hepatocellular carcinoma	miR-518d	Cell proliferation and escape from apoptosis	[168]
56	Hepatocellular carcinoma	miR-215	Cell proliferation and escape from apoptosis	[168]
57	Hepatocellular carcinoma	miR-376a	Cell proliferation and escape from apoptosis	[168]
58	Hepatocellular carcinoma	miR-133b	Cell proliferation and escape from apoptosis	[168]
59	Hepatocellular carcinoma	miR-367	Cell proliferation and escape from apoptosis	[168]
60	Hepatocellular carcinoma	Vasorin	Sustaining of angiogenesis	[169]
61	Hepatocellular carcinoma	miR-103	Sustaining of angiogenesis	[167]
62	Hepatocellular carcinoma	miR-21	Evade immune response and promote inflammation	[115]
63	Leukemia	miR-20b	Cell proliferation and escape from apoptosis	[68]
64	Liposarcoma	miR-25-3p	Evade immune response and promote inflammation	[123]
65	Liposarcoma	miR-92a-3p	Evade immune response and promote inflammation	[123]
66	Lung cancer	miR-143-3p	Sustaining of angiogenesis	[170]
67	Lung cancer	miR-145-5p	Sustaining of angiogenesis	[170]
68	Lung cancer	miR-23a	Sustaining of angiogenesis	[171]
69	Lung cancer	miR-142-3p	Evade immune response and promote inflammation	[117]
70	Melanoma	PGDRF- β	Cell proliferation and escape from apoptosis	[100]
71	Melanoma	Programmed cell death protein 1 and cytotoxic T lymphocyte associated antigen-4	Cell proliferation and escape from apoptosis	[172]
72	Melanoma	ALK isoform	Transfer mutations	[173]
73	Melanoma	miR-155-5p	Evade immune response and promote inflammation	[174]
74	Multiple myeloma	piRNA-823	Sustaining of angiogenesis	[175]
75	Nasopharyngeal carcinoma	miR-23a	Sustaining of angiogenesis	[176]
76	Nasopharyngeal carcinoma	miR-24-3p	Evade immune response and promote inflammation	[177]
77	Nasopharyngeal carcinoma	Galactin-9	Evade immune response and promote inflammation	[178]
78	Oral cancer	miR-142-3p	Sustaining of angiogenesis	[179]
79	Osteosarcoma	miR-675	Evade immune response and promote inflammation	[180]
80	Ovarian cancer	miR-99a-5p	Cell invasion and metastasis	[181]
81	Ovarian cancer	SMAD4	Transfer mutations	[138]
82	Ovarian cancer	miR-1246	Evade immune response and promote inflammation	[121]
83	Ovarian cancer	Arginase-1	Evade immune response and promote inflammation	[182]
84	Ovarian carcinoma	miR-141-3p	Sustaining of angiogenesis	[110]
85	Ovarian serous carcinoma	miR-21	Cell proliferation and escape from apoptosis	[70]
86	Pancreatic cancer	miR-23b-5p	Cell proliferation and escape from apoptosis	[183]
87	Papillary thyroid cancer	miR-146b	Cell proliferation	[184]
88	Papillary thyroid cancer	miR-222	Cell proliferation	[184]
89	Prostate cancer	miR1246	Cell invasion and metastasis	[185]
90	Renal cell carcinoma	Fas ligand	Evade immune response and promote inflammation	[186]

**Table 3:** List of proteins/enzymes from EVs and their role in cancer drug resistance.

S. No.	Protein/Enzyme from EVs	Role	Cancer	Reference
1	Glutathione S-transferases (GSTs)	Use glutathione conjugation to detoxify anticancer drugs	Breast cancer Colon cancer	[217,218]
2	P-gp (MDR1)	Drug efflux	Leukemia Breast cancer Prostate cancer	[203,219,220]
3	TrpC5	Transcriptional activation of the *MDR1* (*ABCB1*) promoter by NFATc3	Breast Cancer (MCF7)	[219]
4	Ezrin	Modulate P-gp	Lung cancer Breast cancer	[221,222,223]
5	Radixin	Modulate P-gp	Colon cancer	[223,224]
6	Moesin	Modulate P-gp	Breast cancer	[223]
7	CD44	Modulate P-gp	Colon cancer Gastric cancer Ovarian cancer Pancreatic cancer	[223,225,226]
8	Multidrug-resistance-associated proteins (MRP1-9/ABCCs)	Drug efflux	Lung cancer Breast cancer Prostate cancer Childhood neuroblastoma	[227,228]
9	ABCG2	Mediates the efficient pumping and concentration of multiple cytotoxic agents	Breast cancer	[229]
10	Galectin-3	Activation of NF-*κ*B	Neck cancer Thyroid cancer Gastric cancer Colon cancer Uterine cancer Renal cancer	[230]
11	Carbonic anhydrase XII (CA XII)	Co-expressed and co-located with P-gp	Renal cancer Breast cancer	[231]
12	UCH-L1	Upregulate P-gp protein expression levels via the MAPK/ERK signaling pathway	Breast cancer	[232]

## Data Availability

Not applicable.

## References

[1] D’Arcy M.S. (2019). Cell death: A review of the major forms of apoptosis, necrosis and autophagy. Cell Biol. Int..

[2] Chaffer C.L., Weinberg R.A. (2011). A perspective on cancer cell metastasis. Science.

[3] Fialkow P.J. (1976). Clonal origin of human tumors. Biochim. Biophys. Acta Rev. Cancer.

[4] Ribatti D., Vacca A. (2008). Overview of angiogenesis during tumor growth. Angiogenesis.

[5] Klaus A., Birchmeier W. (2008). Wnt signalling and its impact on development and cancer. Nat. Rev. Cancer.

[6] Carnino J.M., Hao Kwok Z., Jin Y. (2021). Extracellular Vesicles: A Novel Opportunity for Precision Medicine in Respiratory Diseases. Front. Med..

[7] Xavier C.P., Caires H.R., Barbosa M.A., Bergantim R., Guimarães J.E., Vasconcelos M.H. (2020). The role of extracellular vesicles in the hallmarks of cancer and drug resistance. Cells.

[8] Möller A., Lobb R.J. (2020). The evolving translational potential of small extracellular vesicles in cancer. Nat. Rev. Cancer.

[9] Bongiovanni L., Andriessen A., Wauben M.H., Hoen E.N.N.-T., de Bruin A. (2021). Extracellular Vesicles: Novel Opportunities to Understand and Detect Neoplastic Diseases. Vet. Pathol..

[10] Yamamoto T., Kosaka N., Ochiya T. (2019). Latest advances in extracellular vesicles: From bench to bedside. Sci. Technol. Adv. Mater..

[11] Qian Z., Shen Q., Yang X., Qiu Y., Zhang W. (2015). The role of extracellular vesicles: An epigenetic view of the cancer microenvironment. BioMed Res. Int..

[12] Guescini M., Genedani S., Stocchi V., Agnati L.F. (2010). Astrocytes and Glioblastoma cells release exosomes carrying mtDNA. J. Neural Transm..

[13] O’Loghlen A. (2018). Role for extracellular vesicles in the tumour microenvironment. Philos. Trans. R. Soc. B Biol. Sci..

[14] Harding C., Stahl P. (1983). Transferrin recycling in reticulocytes: pH and iron are important determinants of ligand binding and processing. Biochem. Biophys. Res. Commun..

[15] Pan B.-T., Johnstone R.M. (1983). Fate of the transferrin receptor during maturation of sheep reticulocytes in vitro: Selective externalization of the receptor. Cell.

[16] Harding C.V., Heuser J.E., Stahl P.D. (2013). Exosomes: Looking back three decades and into the future. J. Cell Biol..

[17] Trams E.G., Lauter C.J., Salem J.N., Heine U. (1981). Exfoliation of membrane ecto-enzymes in the form of micro-vesicles. Biochim. Biophys. Acta Biomembr..

[18] Mitchell P., Petfalski E., Shevchenko A., Mann M., Tollervey D. (1997). The exosome: A conserved eukaryotic RNA processing complex containing multiple 3′→ 5′ exoribonucleases. Cell.

[19] Harding C., Heuser J., Stahl P. (1984). Endocytosis and intracellular processing of transferrin and colloidal gold-transferrin in rat reticulocytes: Demonstration of a pathway for receptor shedding. Eur. J. Cell Biol..

[20] Pan B.-T., Teng K., Wu C., Adam M., Johnstone R.M. (1985). Electron microscopic evidence for externalization of the transferrin receptor in vesicular form in sheep reticulocytes. J. Cell Biol..

[21] Raposo G., Nijman H.W., Stoorvogel W., Liejendekker R., Harding C.V., Melief C., Geuze H.J. (1996). B lymphocytes secrete antigen-presenting vesicles. J. Exp. Med..

[22] Zhang H.-G., Grizzle W.E. (2011). Exosomes and cancer: A newly described pathway of immune suppression. Clin. Cancer Res..

[23] Simons M., Raposo G. (2009). Exosomes–vesicular carriers for intercellular communication. Curr. Opin. Cell Biol..

[24] Théry C., Ostrowski M., Segura E. (2009). Membrane vesicles as conveyors of immune responses. Nat. Rev. Immunol..

[25] Chik F., Szyf M., Rabbani S.A. (2011). Role of epigenetics in cancer initiation and progression. Hum. Cell Transform..

[26] Gould S.J., Raposo G. (2013). As we wait: Coping with an imperfect nomenclature for extracellular vesicles. J. Extracell. Vesicles.

[27] Minciacchi V.R., Freeman M.R., Di Vizio D. (2015). Extracellular vesicles in cancer: Exosomes, microvesicles and the emerging role of large oncosomes. Semin. Cell Dev. Biol..

[28] Whiteside T.L. (2018). The potential of tumor-derived exosomes for noninvasive cancer monitoring: An update. Expert Rev. Mol. Diagn..

[29] Samanta S., Rajasingh S., Drosos N., Zhou Z., Dawn B., Rajasingh J. (2018). Exosomes: New molecular targets of diseases. Acta Pharmacol. Sin..

[30] Yáñez-Mó M., Siljander P.R.-M., Andreu Z., Bedina Zavec A., Borràs F.E., Buzas E.I., Buzas K., Casal E., Cappello F., Carvalho J. (2015). Biological properties of extracellular vesicles and their physiological functions. J. Extracell. Vesicles.

[31] Ciardiello C., Cavallini L., Spinelli C., Yang J., Reis-Sobreiro M., De Candia P., Minciacchi V.R., Di Vizio D. (2016). Focus on extracellular vesicles: New frontiers of cell-to-cell communication in cancer. Int. J. Mol. Sci..

[32] Gulinelli S., Salaro E., Vuerich M., Bozzato D., Pizzirani C., Bolognesi G., Idzko M., Virgilio F.D., Ferrari D. (2012). IL-18 associates to microvesicles shed from human macrophages by a LPS/TLR-4 independent mechanism in response to P2X receptor stimulation. Eur. J. Immunol..

[33] Zhang H.-G., Liu C., Su K., Yu S., Zhang L., Zhang S., Wang J., Cao X., Grizzle W., Kimberly R.P. (2006). A membrane form of TNF-α presented by exosomes delays T cell activation-induced cell death. J. Immunol..

[34] Wang G.-J., Liu Y., Qin A., Shah S.V., Deng Z.-b., Xiang X., Cheng Z., Liu C., Wang J., Zhang L. (2008). Thymus exosomes-like particles induce regulatory T cells. J. Immunol..

[35] Miyado K., Yoshida K., Yamagata K., Sakakibara K., Okabe M., Wang X., Miyamoto K., Akutsu H., Kondo T., Takahashi Y. (2008). The fusing ability of sperm is bestowed by CD9-containing vesicles released from eggs in mice. Proc. Natl. Acad. Sci. USA.

[36] Bianchi E., Doe B., Goulding D., Wright G.J. (2014). Juno is the egg Izumo receptor and is essential for mammalian fertilization. Nature.

[37] Desrochers L.M., Bordeleau F., Reinhart-King C.A., Cerione R.A., Antonyak M.A. (2016). Microvesicles provide a mechanism for intercellular communication by embryonic stem cells during embryo implantation. Nat. Commun..

[38] Admyre C., Bohle B., Johansson S.M., Focke-Tejkl M., Valenta R., Scheynius A., Gabrielsson S. (2007). B cell–derived exosomes can present allergen peptides and activate allergen-specific T cells to proliferate and produce TH2-like cytokines. J. Allergy Clin. Immunol..

[39] Montecalvo A., Larregina A.T., Shufesky W.J., Beer Stolz D., Sullivan M.L., Karlsson J.M., Baty C.J., Gibson G.A., Erdos G., Wang Z. (2012). Mechanism of transfer of functional microRNAs between mouse dendritic cells via exosomes. Blood J. Am. Soc. Hematol..

[40] Okoye I.S., Coomes S.M., Pelly V.S., Czieso S., Papayannopoulos V., Tolmachova T., Seabra M.C., Wilson M.S. (2014). MicroRNA-40. containing T-regulatory-cell-derived exosomes suppress pathogenic T helper 1 cells. Immunity.

[41] Lachenal G., Pernet-Gallay K., Chivet M., Hemming F.J., Belly A., Bodon G., Blot B., Haase G., Goldberg Y., Sadoul R. (2011). Release of exosomes from differentiated neurons and its regulation by synaptic glutamatergic activity. Mol. Cell. Neurosci..

[42] Morel L., Regan M., Higashimori H., Ng S.K., Esau C., Vidensky S., Rothstein J., Yang Y. (2013). Neuronal exosomal miRNA-dependent translational regulation of astroglial glutamate transporter GLT1. J. Biol. Chem..

[43] Fröhlich D., Kuo W.P., Frühbeis C., Sun J.-J., Zehendner C.M., Luhmann H.J., Pinto S., Toedling J., Trotter J., Krämer-Albers E.-M. (2014). Multifaceted effects of oligodendroglial exosomes on neurons: Impact on neuronal firing rate, signal transduction and gene regulation. Philos. Trans. R. Soc. B Biol. Sci..

[44] Bakhti M., Winter C., Simons M. (2011). Inhibition of myelin membrane sheath formation by oligodendrocyte-derived exosome-like vesicles. J. Biol. Chem..

[45] Lopez-Verrilli M.A., Picou F., Court F.A. (2013). Schwann cell-derived exosomes enhance axonal regeneration in the peripheral nervous system. Glia.

[46] Antonucci F., Turola E., Riganti L., Caleo M., Gabrielli M., Perrotta C., Novellino L., Clementi E., Giussani P., Viani P. (2012). Microvesicles released from microglia stimulate synaptic activity via enhanced sphingolipid metabolism. EMBO J..

[47] Biro E., Sturk-Maquelin K., Vogel G., Meuleman D., Smit M., Hack C., Sturk A., Nieuwland R. (2003). Human cell-derived microparticles promote thrombus formation in vivo in a tissue factor-dependent manner. J. Thromb. Haemost..

[48] Taraboletti G., D’Ascenzo S., Borsotti P., Giavazzi R., Pavan A., Dolo V. (2002). Shedding of the matrix metalloproteinases MMP-2, MMP-9, and MT1-MMP as membrane vesicle-associated components by endothelial cells. Am. J. Pathol..

[49] Brill A., Dashevsky O., Rivo J., Gozal Y., Varon D. (2005). Platelet-derived microparticles induce angiogenesis and stimulate post-ischemic revascularization. Cardiovasc. Res..

[50] Mu W., Rana S., Zöller M. (2013). Host matrix modulation by tumor exosomes promotes motility and invasiveness. Neoplasia.

[51] Ma Z., Wang Y., Li H. (2020). Applications of extracellular vesicles in tissue regeneration. Biomicrofluidics.

[52] Klymenko Y., Nephew K.P. (2018). Epigenetic Crosstalk between the Tumor Microenvironment and Ovarian Cancer Cells: A Therapeutic Road Less Traveled. Cancers (Basel).

[53] Jahanban-Esfahlan R., Seidi K., Monhemi H., Adli A.D.F., Minofar B., Zare P., Farajzadeh D., Farajnia S., Behzadi R., Abbasi M.M. (2017). RGD delivery of truncated coagulase to tumor vasculature affords local thrombotic activity to induce infarction of tumors in mice. Sci. Rep..

[54] Baghban R., Roshangar L., Jahanban-Esfahlan R., Seidi K., Ebrahimi-Kalan A., Jaymand M., Kolahian S., Javaheri T., Zare P. (2020). Tumor microenvironment complexity and therapeutic implications at a glance. Cell Commun. Signal..

[55] Hanahan D., Coussens L.M. (2012). Accessories to the crime: Functions of cells recruited to the tumor microenvironment. Cancer Cell.

[56] Frisch J., Angenendt A., Hoth M., Prates Roma L., Lis A. (2019). STIM-Orai channels and reactive oxygen species in the tumor microenvironment. Cancers.

[57] Zhang D.X., Vu L.T., Ismail N.N., Le M.T., Grimson A. (2021). Landscape of extracellular vesicles in the tumour microenvironment: Interactions with stromal cells and with non-cell components, and impacts on metabolic reprogramming, horizontal transfer of neoplastic traits, and the emergence of therapeutic resistance. Semin. Cancer Biol..

[58] Berger S.L., Kouzarides T., Shiekhattar R., Shilatifard A. (2009). An operational definition of epigenetics. Genes Dev..

[59] Fouse S.D., Costello J.F. (2009). Epigenetics of neurological cancers. Future Oncol..

[60] Moran B., Silva R., Perry A.S., Gallagher W.M. (2018). Epigenetics of malignant melanoma. Semin. Cancer Biol..

[61] Sarkar D., Leung E.Y., Baguley B.C., Finlay G.J., Askarian-Amiri M.E. (2015). Epigenetic regulation in human melanoma: Past and future. Epigenetics.

[62] Micevic G., Theodosakis N., Bosenberg M. (2017). Aberrant DNA methylation in melanoma: Biomarker and therapeutic opportunities. Clin. Epigenetics.

[63] Peng B., Hodge D.R., Thomas S.B., Cherry J.M., Munroe D.J., Pompeia C., Xiao W., Farrar W.L. (2005). Epigenetic silencing of the human nucleotide excision repair gene, hHR23B, in interleukin-6-responsive multiple myeloma KAS-6/1 cells. J. Biol. Chem..

[64] Maio M., Covre A., Fratta E., Di Giacomo A.M., Taverna P., Natali P.G., Coral S., Sigalotti L. (2015). Molecular pathways: At the crossroads of cancer epigenetics and immunotherapy. Clin. Cancer Res..

[65] Siebenkäs C., Chiappinelli K.B., Guzzetta A.A., Sharma A., Jeschke J., Vatapalli R., Baylin S.B., Ahuja N. (2020). Correction: Inhibiting DNA methylation activates cancer testis antigens and expression of the antigen processing and presentation machinery in colon and ovarian cancer cells. PLoS ONE.

[66] Luo N., Nixon M.J., Gonzalez-Ericsson P.I., Sanchez V., Opalenik S.R., Li H., Zahnow C.A., Nickels M.L., Liu F., Tantawy M.N. (2018). DNA methyltransferase inhibition upregulates MHC-I to potentiate cytotoxic T lymphocyte responses in breast cancer. Nat. Commun..

[67] Ratajczak J., Wysoczynski M., Hayek F., Janowska-Wieczorek A., Ratajczak M. (2006). Membrane-derived microvesicles: Important and underappreciated mediators of cell-to-cell communication. Leukemia.

[68] Lu L., Chen X., Tao H., Xiong W., Jie S., Li H. (2015). Regulation of the expression of zinc finger protein genes by microRNAs enriched within acute lymphoblastic leukemia-derived microvesicles. Genet. Mol. Res..

[69] Liu M.X., Juan L., Ming X., Gao Z.K., Wang X.H., Zhang Y., Shang M.H., Yin L.H., Pu Y.P., Ran L. (2018). miR-93-5p transferred by exosomes promotes the proliferation of esophageal cancer cells via intercellular communication by targeting PTEN. Biomed. Environ. Sci..

[70] Cappellesso R., Tinazzi A., Giurici T., Simonato F., Guzzardo V., Ventura L., Crescenzi M., Chiarelli S., Fassina A. (2014). Programmed cell death 4 and micro RNA 21 inverse expression is maintained in cells and exosomes from ovarian serous carcinoma effusions. Cancer Cytopathol..

[71] Goyal B., Yadav S.R.M., Awasthee N., Gupta S., Kunnumakkara A.B., Gupta S.C. (2021). Diagnostic, prognostic, and therapeutic significance of long non-coding RNA MALAT1 in cancer. Biochim. Biophys. Acta Rev. Cancer.

[72] Brockdorff N. (2013). Noncoding RNA and Polycomb recruitment. RNA.

[73] Mishra S., Verma S.S., Rai V., Awasthee N., Chava S., Hui K.M., Kumar A.P., Challagundla K.B., Sethi G., Gupta S.C. (2019). Long non-coding RNAs are emerging targets of phytochemicals for cancer and other chronic diseases. Cell. Mol. Life Sci..

[74] Kogure T., Yan I.K., Lin W.-L., Patel T. (2013). Extracellular vesicle–mediated transfer of a novel long noncoding RNA TUC339: A mechanism of intercellular signaling in human hepatocellular cancer. Genes Cancer.

[75] Pan L., Liang W., Fu M., Huang Z.-h., Li X., Zhang W., Zhang P., Qian H., Jiang P.-C., Xu W.-R. (2017). Exosomes-mediated transfer of long noncoding RNA ZFAS1 promotes gastric cancer progression. J. Cancer Res. Clin. Oncol..

[76] Chen G., Huang A.C., Zhang W., Zhang G., Wu M., Xu W., Yu Z., Yang J., Wang B., Sun H. (2018). Exosomal PD-L1 contributes to immunosuppression and is associated with anti-PD-1 response. Nature.

[77] Cao Y.L., Zhuang T., Xing B.H., Li N., Li Q. (2017). Exosomal DNMT1 mediates cisplatin resistance in ovarian cancer. Cell Biochem. Funct..

[78] Yang S.-J., Wang D.-D., Li J., Xu H.-Z., Shen H.-Y., Chen X., Zhou S.-Y., Zhong S.-L., Zhao J.-H., Tang J.-H. (2017). Predictive role of GSTP1-containing exosomes in chemotherapy-resistant breast cancer. Gene.

[79] Sousa D., Lima R.T., Vasconcelos M.H. (2015). Intercellular transfer of cancer drug resistance traits by extracellular vesicles. Trends Mol. Med..

[80] Skog J., Würdinger T., Van Rijn S., Meijer D.H., Gainche L., Curry W.T., Carter B.S., Krichevsky A.M., Breakefield X.O. (2008). Glioblastoma microvesicles transport RNA and proteins that promote tumour growth and provide diagnostic biomarkers. Nat. Cell Biol..

[81] Taylor D.D., Gercel-Taylor C. (2008). MicroRNA signatures of tumor-derived exosomes as diagnostic biomarkers of ovarian cancer. Gynecol. Oncol..

[82] De Souza P.S., Cruz A.L., Viola J.P., Maia R.C. (2015). Microparticles induce multifactorial resistance through oncogenic pathways independently of cancer cell type. Cancer Sci..

[83] Wu H., Zhou J., Mei S., Wu D., Mu Z., Chen B., Xie Y., Ye Y., Liu J. (2017). Circulating exosomal microRNA-96 promotes cell proliferation, migration and drug resistance by targeting LMO7. J. Cell. Mol. Med..

[84] Yu D.-D., Wu Y., Zhang X.-H., Lv M.-M., Chen W.-X., Chen X., Yang S.-J., Shen H., Zhong S.-L., Tang J.-H. (2016). Exosomes from adriamycin-resistant breast cancer cells transmit drug resistance partly by delivering miR-222. Tumor Biol..

[85] Corcoran C., Friel A.M., Duffy M.J., Crown J., O’Driscoll L. (2011). Intracellular and extracellular microRNAs in breast cancer. Clin. Chem..

[86] Wei Y., Lai X., Yu S., Chen S., Ma Y., Zhang Y., Li H., Zhu X., Yao L., Zhang J. (2014). Exosomal miR-221/222 enhances tamoxifen resistance in recipient ER-positive breast cancer cells. Breast Cancer Res. Treat..

[87] Gezer U., Özgür E., Cetinkaya M., Isin M., Dalay N. (2014). Long non-coding RNAs with low expression levels in cells are enriched in secreted exosomes. Cell Biol. Int..

[88] Biswas S., Guix M., Rinehart C., Dugger T.C., Chytil A., Moses H.L., Freeman M.L., Arteaga C.L. (2007). Inhibition of TGF-β with neutralizing antibodies prevents radiation-induced acceleration of metastatic cancer progression. J. Clin. Investig..

[89] Takahashi K., Yan I.K., Kogure T., Haga H., Patel T. (2014). Extracellular vesicle-mediated transfer of long non-coding RNA ROR modulates chemosensitivity in human hepatocellular cancer. FEBS Open Bio.

[90] Takahashi K., Yan I.K., Wood J., Haga H., Patel T. (2014). Involvement of extracellular vesicle long noncoding RNA (linc-VLDLR) in tumor cell responses to chemotherapy. Mol. Cancer Res..

[91] Qu L., Ding J., Chen C., Wu Z.-J., Liu B., Gao Y., Chen W., Liu F., Sun W., Li X.-F. (2016). Exosome-transmitted lncARSR promotes sunitinib resistance in renal cancer by acting as a competing endogenous RNA. Cancer Cell.

[92] Wang J., Lv B., Su Y., Wang X., Bu J., Yao L. (2019). Exosome-mediated transfer of lncRNA HOTTIP promotes cisplatin resistance in gastric cancer cells by regulating HMGA1/miR-218 axis. OncoTargets Ther..

[93] Stoner S.A., Duggan E., Condello D., Guerrero A., Turk J.R., Narayanan P.K., Nolan J.P. (2016). High sensitivity flow cytometry of membrane vesicles. Cytom. Part A.

[94] De Lellis L., Florio R., Di Bella M.C., Brocco D., Guidotti F., Tinari N., Grassadonia A., Lattanzio R., Cama A., Veschi S. (2021). Exosomes as pleiotropic players in pancreatic cancer. Biomedicines.

[95] Siveen K.S., Raza A., Ahmed E.I., Khan A.Q., Prabhu K.S., Kuttikrishnan S., Mateo J.M., Zayed H., Rasul K., Azizi F. (2019). The role of extracellular vesicles as modulators of the tumor microenvironment, metastasis and drug resistance in colorectal cancer. Cancers.

[96] Rackov G., Garcia-Romero N., Esteban-Rubio S., Carrión-Navarro J., Belda-Iniesta C., Ayuso-Sacido A. (2018). Vesicle-mediated control of cell function: The role of extracellular matrix and microenvironment. Front. Physiol..

[97] Tang T.-T., Wang B., Wu M., Li Z.-L., Feng Y., Cao J.-Y., Yin D., Liu H., Tang R.-N., Crowley S.D. (2020). Extracellular vesicle–encapsulated IL-10 as novel nanotherapeutics against ischemic AKI. Sci. Adv..

[98] Arkhypov I., Lasser S., Petrova V., Weber R., Groth C., Utikal J., Altevogt P., Umansky V. (2020). Myeloid cell modulation by tumor-derived extracellular vesicles. Int. J. Mol. Sci..

[99] Pavlyukov M.S., Yu H., Bastola S., Minata M., Shender V.O., Lee Y., Zhang S., Wang J., Komarova S., Wang J. (2018). Apoptotic cell-derived extracellular vesicles promote malignancy of glioblastoma via intercellular transfer of splicing factors. Cancer Cell.

[100] Vella L.J., Behren A., Coleman B., Greening D.W., Hill A.F., Cebon J. (2017). Intercellular resistance to BRAF inhibition can be mediated by extracellular vesicle–associated PDGFRβ. Neoplasia.

[101] Brzozowski J.S., Bond D.R., Jankowski H., Goldie B.J., Burchell R., Naudin C., Smith N.D., Scarlett C.J., Larsen M.R., Dun M.D. (2018). Extracellular vesicles with altered tetraspanin CD9 and CD151 levels confer increased prostate cell motility and invasion. Sci. Rep..

[102] Fu Q., Zhang Q., Lou Y., Yang J., Nie G., Chen Q., Chen Y., Zhang J., Wang J., Wei T. (2019). Correction: Primary tumor-derived exosomes facilitate metastasis by regulating adhesion of circulating tumor cells via SMAD3 in liver cancer. Oncogene.

[103] Dörsam B., Bösl T., Reiners K.S., Barnert S., Schubert R., Shatnyeva O., Zigrino P., Engert A., Hansen H.P., von Strandmann E.P. (2018). Hodgkin lymphoma-derived extracellular vesicles change the secretome of fibroblasts toward a CAF phenotype. Front. Immunol..

[104] Ning X., Zhang H., Wang C., Song X. (2018). Exosomes released by gastric cancer cells induce transition of pericytes into cancer-associated 104. fibroblasts. Med. Sci. Monit. Int. Med. J. Exp. Clin. Res..

[105] Zhou W., Fong M.Y., Min Y., Somlo G., Liu L., Palomares M.R., Yu Y., Chow A., O’Connor S.T.F., Chin A.R. (2014). Cancer-secreted miR-105 destroys vascular endothelial barriers to promote metastasis. Cancer Cell.

[106] Adair T.H., Montani J.-P. (2010). Angiogenesis. Proceedings of the Colloquium Series on Integrated Systems Physiology: From Molecule to Function.

[107] Nazarenko I., Rana S., Baumann A., McAlear J., Hellwig A., Trendelenburg M., Lochnit G., Preissner K.T., Zöller M. (2010). Cell surface tetraspanin Tspan8 contributes to molecular pathways of exosome-induced endothelial cell activation. Cancer Res..

[108] Zhuang G., Wu X., Jiang Z., Kasman I., Yao J., Guan Y., Oeh J., Modrusan Z., Bais C., Sampath D. (2012). Tumour-secreted miR-9 promotes endothelial cell migration and angiogenesis by activating the JAK-STAT pathway. EMBO J..

[109] Li B., Hong J., Hong M., Wang Y., Yu T., Zang S., Wu Q. (2019). piRNA-823 delivered by multiple myeloma-derived extracellular vesicles promoted tumorigenesis through re-educating endothelial cells in the tumor environment. Oncogene.

[110] Masoumi-Dehghi S., Babashah S., Sadeghizadeh M. (2020). microRNA-141-3p-containing small extracellular vesicles derived from epithelial ovarian cancer cells promote endothelial cell angiogenesis through activating the JAK/STAT3 and NF-κB signaling pathways. J. Cell Commun. Signal..

[111] Kosaka N., Iguchi H., Hagiwara K., Yoshioka Y., Takeshita F., Ochiya T. (2013). Neutral sphingomyelinase 2 (nSMase2)-dependent exosomal transfer of angiogenic microRNAs regulate cancer cell metastasis. J. Biol. Chem..

[112] Kosaka N., Yoshioka Y., Fujita Y., Ochiya T. (2016). Versatile roles of extracellular vesicles in cancer. J. Clin. Investig..

[113] Cano R.L.E., Lopera H.D.E. (2013). Introduction to T and B lymphocytes. Autoimmunity: From Bench to Bedside.

[114] Giusti I., Di Francesco M., D’Ascenzo S., Palmerini M.G., Macchiarelli G., Carta G., Dolo V. (2018). Ovarian cancer-derived extracellular vesicles affect normal human fibroblast behavior. Cancer Biol. Ther..

[115] Zhou Y., Ren H., Dai B., Li J., Shang L., Huang J., Shi X. (2018). Hepatocellular carcinoma-derived exosomal miRNA-21 contributes to tumor progression by converting hepatocyte stellate cells to cancer-associated fibroblasts. J. Exp. Clin. Cancer Res..

[116] Pang W., Su J., Wang Y., Feng H., Dai X., Yuan Y., Chen X., Yao W. (2015). Pancreatic cancer-secreted miR-155 implicates in the conversion from normal fibroblasts to cancer-associated fibroblasts. Cancer Sci..

[117] Lawson J., Dickman C., Towle R., Jabalee J., Javer A., Garnis C. (2019). Extracellular vesicle secretion of miR-142-3p from lung adenocarcinoma cells induces tumor promoting changes in the stroma through cell-cell communication. Mol. Carcinog..

[118] Snyder A., Makarov V., Merghoub T., Yuan J., Zaretsky J.M., Desrichard A., Walsh L.A., Postow M.A., Wong P., Ho T.S. (2014). Genetic basis for clinical response to CTLA-4 blockade in melanoma. N. Engl. J. Med..

[119] Topalian S.L., Hodi F.S., Brahmer J.R., Gettinger S.N., Smith D.C., McDermott D.F., Powderly J.D., Carvajal R.D., Sosman J.A., Atkins M.B. (2012). Safety, activity, and immune correlates of anti–PD-1 antibody in cancer. N. Engl. J. Med..

[120] Pucci F., Garris C., Lai C.P., Newton A., Pfirschke C., Engblom C., Alvarez D., Sprachman M., Evavold C., Magnuson A. (2016). SCS macrophages suppress melanoma by restricting tumor-derived vesicle–B cell interactions. Science.

[121] Kanlikilicer P., Bayraktar R., Denizli M., Rashed M.H., Ivan C., Aslan B., Mitra R., Karagoz K., Bayraktar E., Zhang X. (2018). Exosomal miRNA confers chemo resistance via targeting Cav1/p-gp/M2-type macrophage axis in ovarian cancer. EBioMedicine.

[122] Cooks T., Pateras I.S., Jenkins L.M., Patel K.M., Robles A.I., Morris J., Forshew T., Appella E., Gorgoulis V.G., Harris C.C. (2018). Mutant p53 cancers reprogram macrophages to tumor supporting macrophages via exosomal miR-1246. Nat. Commun..

[123] Casadei L., Calore F., Creighton C.J., Guescini M., Batte K., Iwenofu O.H., Zewdu A., Braggio D.A., Bill K.L., Fadda P. (2017). Exosome-derived miR-25-3p and miR-92a-3p stimulate liposarcoma progression. Cancer Res..

[124] Chen X., Zhou J., Li X., Wang X., Lin Y., Wang X. (2018). Exosomes derived from hypoxic epithelial ovarian cancer cells deliver microRNAs to macrophages and elicit a tumor-promoted phenotype. Cancer Lett..

[125] Van der Vos K.E., Abels E.R., Zhang X., Lai C., Carrizosa E., Oakley D., Prabhakar S., Mardini O., Crommentuijn M.H., Skog J. (2015). Directly visualized glioblastoma-derived extracellular vesicles transfer RNA to microglia/macrophages in the brain. Neuro-Oncology.

[126] Viaud S., Terme M., Flament C., Taieb J., Andre F., Novault S., Escudier B., Robert C., Caillat-Zucman S., Tursz T. (2009). Dendritic cell-derived exosomes promote natural killer cell activation and proliferation: A role for NKG2D ligands and IL-15Rα. PLoS ONE.

[127] Zhang X., Yuan X., Shi H., Wu L., Qian H., Xu W. (2015). Exosomes in cancer: Small particle, big player. J. Hematol. Oncol..

[128] Czernek L., Düchler M. (2017). Functions of cancer-derived extracellular vesicles in immunosuppression. Arch. Immunol. Ther. Exp..

[129] Szczepanski M.J., Szajnik M., Welsh A., Whiteside T.L., Boyiadzis M. (2011). Blast-derived microvesicles in sera from patients with acute myeloid leukemia suppress natural killer cell function via membrane-associated transforming growth factor-β1. Haematologica.

[130] Bubeník J. (2004). MHC class I down-regulation: Tumour escape from immune surveillance?. Int. J. Oncol..

[131] Ljunggren H.-G., Kärre K. (1990). In search of the ‘missing self’: MHC molecules and NK cell recognition. Immunol. Today.

[132] Clayton A., Mitchell J.P., Linnane S., Mason M.D., Tabi Z. (2008). Human tumor-derived exosomes down-modulate NKG2D expression. J. Immunol..

[133] Hedlund M., Nagaeva O., Kargl D., Baranov V., Mincheva-Nilsson L. (2011). Thermal-and oxidative stress causes enhanced release of NKG2D ligand-bearing immunosuppressive exosomes in leukemia/lymphoma T and B cells. PLoS ONE.

[134] Dörsam B., Reiners K.S., von Strandmann E.P. (2018). Cancer-derived extracellular vesicles: Friend and foe of tumour immunosurveillance. Philos. Trans. R. Soc. B Biol. Sci..

[135] Viaud S., Théry C., Ploix S., Tursz T., Lapierre V., Lantz O., Zitvogel L., Chaput N. (2010). Dendritic cell-derived exosomes for cancer immunotherapy: What’s next?. Cancer Res..

[136] Lamparski H.G., Metha-Damani A., Yao J.-Y., Patel S., Hsu D.-H., Ruegg C., Le Pecq J.-B. (2002). Production and characterization of clinical grade exosomes derived from dendritic cells. J. Immunol. Methods.

[137] Zeng A., Yan W., Liu Y., Wang Z., Hu Q., Nie E., Zhou X., Li R., Wang X., Jiang T. (2017). Tumour exosomes from cells harbouring PTPRZ1–MET fusion contribute to a malignant phenotype and temozolomide chemoresistance in glioblastoma. Oncogene.

[138] Crow J., Atay S., Banskota S., Artale B., Schmitt S., Godwin A.K. (2017). Exosomes as mediators of platinum resistance in ovarian cancer. Oncotarget.

[139] Li X.J., Ren Z.J., Tang J.H., Yu Q. (2017). Exosomal MicroRNA MiR-1246 promotes cell proliferation, invasion and drug resistance by targeting CCNG2 in breast cancer. Cell. Physiol. Biochem..

[140] Gregory P.A., Bert A.G., Paterson E.L., Barry S.C., Tsykin A., Farshid G., Vadas M.A., Khew-Goodall Y., Goodall G.J. (2008). The miR-200 family and miR-205 regulate epithelial to mesenchymal transition by targeting ZEB1 and SIP1. Nat. Cell Biol..

[141] Campos A., Salomon C., Bustos R., Díaz J., Martínez S., Silva V., Reyes C., Díaz-Valdivia N., Varas-Godoy M., Lobos-González L. (2018). Caveolin-1-containing extracellular vesicles transport adhesion proteins and promote malignancy in breast cancer cell lines. Nanomedicine.

[142] Fong M.Y., Zhou W., Liu L., Alontaga A.Y., Chandra M., Ashby J., Chow A., O’Connor S.T.F., Li S., Chin A.R. (2015). Breast-cancer-secreted miR-122 reprograms glucose metabolism in premetastatic niche to promote metastasis. Nat. Cell Biol..

[143] Kitdumrongthum S., Metheetrairut C., Charoensawan V., Ounjai P., Janpipatkul K., Panvongsa W., Weerachayaphorn J., Piyachaturawat P., Chairoungdua A. (2018). Dysregulated microRNA expression profiles in cholangiocarcinoma cell-derived exosomes. Life Sci..

[144] Teng Y., Ren Y., Hu X., Mu J., Samykutty A., Zhuang X., Deng Z., Kumar A., Zhang L., Merchant M.L. (2017). MVP-mediated exosomal sorting of miR-193a promotes colon cancer progression. Nat. Commun..

[145] Zhang Z., Xing T., Chen Y., Xiao J. (2018). Exosome-mediated miR-200b promotes colorectal cancer proliferation upon TGF-β1 exposure. Biomed. Pharmacother..

[146] Zeng Z., Li Y., Pan Y., Lan X., Song F., Sun J., Zhou K., Liu X., Ren X., Wang F. (2018). Cancer-derived exosomal miR-25-3p promotes pre-metastatic niche formation by inducing vascular permeability and angiogenesis. Nat. Commun..

[147] Harada T., Yamamoto H., Kishida S., Kishida M., Awada C., Takao T., Kikuchi A. (2017). Wnt5b-associated exosomes promote cancer cell migration and proliferation. Cancer Sci..

[148] Higginbotham J.N., Beckler M.D., Gephart J.D., Franklin J.L., Bogatcheva G., Kremers G.-J., Piston D.W., Ayers G.D., McConnell R.E., Tyska M.J. (2011). Amphiregulin exosomes increase cancer cell invasion. Curr. Biol..

[149] Kalra H., Gangoda L., Fonseka P., Chitti S.V., Liem M., Keerthikumar S., Samuel M., Boukouris S., Al Saffar H., Collins C. (2019). Extracellular vesicles containing oncogenic mutant β-catenin activate Wnt signalling pathway in the recipient cells. J. Extracell. Vesicles.

[150] Muturi H.T., Dreesen J.D., Nilewski E., Jastrow H., Giebel B., Ergun S., Singer B.B. (2013). Tumor and endothelial cell-derived microvesicles carry distinct CEACAMs and influence T-cell behavior. PLoS ONE.

[151] Dai G., Yao X., Zhang Y., Gu J., Geng Y., Xue F., Zhang J. (2018). Colorectal cancer cell–derived exosomes containing miR-10b regulate fibroblast cells via the PI3K/Akt pathway. Bull. Cancer.

[152] Guo K., Yao J., Yu Q., Li Z., Huang H., Cheng J., Wang Z., Zhu Y. (2017). The expression pattern of long non-coding RNA PVT1 in tumor tissues and in extracellular vesicles of colorectal cancer correlates with cancer progression. Tumor Biol..

[153] Zhang Y., Zhou M., Bai L., Han R., Lv K., Wang Z. (2018). Extracellular vesicles promote esophageal cancer progression by delivering lncZEB1-AS1 between cells. Eur. Rev. Med. Pharm. Sci..

[154] Yang H., Fu H., Wang B., Zhang X., Mao J., Li X., Wang M., Sun Z., Qian H., Xu W. (2018). Exosomal miR-423-5p targets SUFU to promote cancer growth and metastasis and serves as a novel marker for gastric cancer. Mol. Carcinog..

[155] Wang J., Guan X., Zhang Y., Ge S., Zhang L., Li H., Wang X., Liu R., Ning T., Deng T. (2018). Exosomal miR-27a derived from gastric cancer cells regulates the transformation of fibroblasts into cancer-associated fibroblasts. Cell. Physiol. Biochem..

[156] Setti M., Osti D., Richichi C., Ortensi B., Del Bene M., Fornasari L., Beznoussenko G., Mironov A., Rappa G., Cuomo A. (2015). Extracellular vesicle-mediated transfer of CLIC1 protein is a novel mechanism for the regulation of glioblastoma growth. Oncotarget.

[157] Lang H.-L., Hu G.-W., Zhang B., Kuang W., Chen Y., Wu L., Xu G.-H. (2017). Glioma cells enhance angiogenesis and inhibit endothelial cell apoptosis through the release of exosomes that contain long non-coding RNA CCAT2. Oncol. Rep..

[158] Lang H., Hu G., Chen Y., Liu Y., Tu W., Lu Y., Wu L., Xu G. (2017). Glioma cells promote angiogenesis through the release of exosomes containing long non-coding RNA POU3F3. Eur. Rev. Med. Pharm. Sci..

[159] Sun X., Ma X., Wang J., Zhao Y., Wang Y., Bihl J.C., Chen Y., Jiang C. (2017). Glioma stem cells-derived exosomes promote the angiogenic ability of endothelial cells through miR-21/VEGF signal. Oncotarget.

[160] Giusti I., Delle Monache S., Di Francesco M., Sanità P., D’Ascenzo S., Gravina G.L., Festuccia C., Dolo V. (2016). From glioblastoma to endothelial cells through extracellular vesicles: Messages for angiogenesis. Tumor. Biol..

[161] Cai Q., Zhu A., Gong L. (2018). Exosomes of glioma cells deliver miR-148a to promote proliferation and metastasis of glioblastoma via targeting CADM1. Bull. Cancer.

[162] Al-Nedawi K., Meehan B., Micallef J., Lhotak V., May L., Guha A., Rak J. (2008). Intercellular transfer of the oncogenic receptor EGFRvIII by microvesicles derived from tumour cells. Nat. Cell Biol..

[163] Ricklefs F.L., Alayo Q., Krenzlin H., Mahmoud A.B., Speranza M.C., Nakashima H., Hayes J.L., Lee K., Balaj L., Passaro C. (2018). Immune evasion mediated by PD-L1 on glioblastoma-derived extracellular vesicles. Sci. Adv..

[164] Sato S., Vasaikar S., Eskaros A., Kim Y., Lewis J.S., Zhang B., Zijlstra A., Weaver A.M. (2019). EPHB2 carried on small extracellular vesicles induces tumor angiogenesis via activation of ephrin reverse signaling. JCI Insight.

[165] Qu Z., Feng J., Pan H., Jiang Y., Duan Y., Fa Z. (2019). Exosomes derived from HCC cells with different invasion characteristics mediated EMT through TGF-β/Smad signaling pathway. OncoTargets Ther..

[166] Xue X., Wang X., Zhao Y., Hu R., Qin L. (2018). Exosomal miR-93 promotes proliferation and invasion in hepatocellular carcinoma by directly inhibiting TIMP2/TP53INP1/CDKN1A. Biochem. Biophys. Res. Commun..

[167] Fang J.H., Zhang Z.J., Shang L.R., Luo Y.W., Lin Y.F., Yuan Y., Zhuang S.M. (2018). Hepatoma cell-secreted exosomal microRNA-103 increases vascular permeability and promotes metastasis by targeting junction proteins. Hepatology.

[168] Kogure T., Lin W.L., Yan I.K., Braconi C., Patel T. (2011). Intercellular nanovesicle-mediated microRNA transfer: A mechanism of environmental modulation of hepatocellular cancer cell growth. Hepatology.

[169] Huang A., Dong J., Li S., Wang C., Ding H., Li H., Su X., Ge X., Sun L., Bai C. (2015). Exosomal transfer of vasorin expressed in hepatocellular carcinoma cells promotes migration of human umbilical vein endothelial cells. Int. J. Biol. Sci..

[170] Lawson J., Dickman C., MacLellan S., Towle R., Jabalee J., Lam S., Garnis C. (2017). Selective secretion of microRNAs from lung cancer cells via extracellular vesicles promotes CAMK1D-mediated tube formation in endothelial cells. Oncotarget.

[171] Zheng Y., Liu L., Chen C., Ming P., Huang Q., Li C., Cao D., Xu X., Ge W. (2017). The extracellular vesicles secreted by lung cancer cells in radiation therapy promote endothelial cell angiogenesis by transferring miR-23a. PeerJ.

[172] Hamid O., Robert C., Daud A., Hodi F.S., Hwu W.-J., Kefford R., Wolchok J.D., Hersey P., Joseph R.W., Weber J.S. (2013). Safety and tumor responses with lambrolizumab (anti–PD-1) in melanoma. N. Engl. J. Med..

[173] Cesi G., Philippidou D., Kozar I., Kim Y.J., Bernardin F., Van Niel G., Wienecke-Baldacchino A., Felten P., Letellier E., Dengler S. (2018). A new ALK isoform transported by extracellular vesicles confers drug resistance to melanoma cells. Mol. Cancer.

[174] Zhou X., Yan T., Huang C., Xu Z., Wang L., Jiang E., Wang H., Chen Y., Liu K., Shao Z. (2018). Melanoma cell-secreted exosomal miR-155-5p induce proangiogenic switch of cancer-associated fibroblasts via SOCS1/JAK2/STAT3 signaling pathway. J. Exp. Clin. Cancer Res..

[175] Yan H., Wu Q.L., Sun C.Y., Ai C.-Y., Deng J., Zhang L., Chen L., Chu Z.-B., Tang B., Wang K. (2015). piRNA-823 contributes to tumorigenesis by regulating de novo DNA methylation and angiogenesis in multiple myeloma. Leukemia.

[176] Bao L., You B., Shi S., Shan Y., Zhang Q., Yue H., Zhang J., Zhang W., Shi Y., Liu Y. (2018). Metastasis-associated miR-23a from nasopharyngeal carcinoma-derived exosomes mediates angiogenesis by repressing a novel target gene TSGA10. Oncogene.

[177] Ye S.-B., Li Z.-L., Luo D.-H., Huang B.-J., Chen Y.-S., Zhang X.-S., Cui J., Zeng Y.-X., Li J. (2014). Tumor-derived exosomes promote tumor progression and T-cell dysfunction through the regulation of enriched exosomal microRNAs in human nasopharyngeal carcinoma. Oncotarget.

[178] Klibi J., Niki T., Riedel A., Pioche-Durieu C., Souquere S., Rubinstein E., Le Moulec S., Guigay J., Hirashima M., Guemira F. (2009). Blood diffusion and Th1-suppressive effects of galectin-9–containing exosomes released by Epstein-Barr virus–infected nasopharyngeal carcinoma cells. Blood J. Am. Soc. Hematol..

[179] Dickman C.T., Lawson J., Jabalee J., MacLellan S.A., LePard N.E., Bennewith K.L., Garnis C. (2017). Selective extracellular vesicle exclusion of miR-142-3p by oral cancer cells promotes both internal and extracellular malignant phenotypes. Oncotarget.

[180] Gong L., Bao Q., Hu C., Wang J., Zhou Q., Wei L., Tong L., Zhang W., Shen Y. (2018). Exosomal miR-675 from metastatic osteosarcoma promotes cell migration and invasion by targeting CALN1. Biochem. Biophys. Res. Commun..

[181] Yoshimura A., Sawada K., Nakamura K., Kinose Y., Nakatsuka E., Kobayashi M., Miyamoto M., Ishida K., Matsumoto Y., Kodama M. (2018). Exosomal miR-99a-5p is elevated in sera of ovarian cancer patients and promotes cancer cell invasion by increasing fibronectin and vitronectin expression in neighboring peritoneal mesothelial cells. BMC Cancer.

[182] Czystowska-Kuzmicz M., Sosnowska A., Nowis D., Ramji K., Szajnik M., Chlebowska-Tuz J., Wolinska E., Gaj P., Grazul M., Pilch Z. (2019). Small extracellular vesicles containing arginase-1 suppress T-cell responses and promote tumor growth in ovarian carcinoma. Nat. Commun..

[183] Chen D., Wu X., Xia M., Wu F., Ding J., Jiao Y., Zhan Q., An F. (2017). Upregulated exosomic miR-23b-3p plays regulatory roles in the progression of pancreatic cancer. Oncol. Rep..

[184] Lee J.C., Zhao J.-T., Gundara J., Serpell J., Bach L.A., Sidhu S. (2015). Papillary thyroid cancer–derived exosomes contain miRNA-146b and miRNA-222. J. Surg. Res..

[185] Bhagirath D., Yang T.L., Bucay N., Sekhon K., Majid S., Shahryari V., Dahiya R., Tanaka Y., Saini S. (2018). microRNA-1246 is an exosomal biomarker for aggressive prostate cancer. Cancer Res..

[186] Yang L., Wu X., Wang D., Luo C., Chen L. (2013). Renal carcinoma cell-derived exosomes induce human immortalized line of Jurkat T lymphocyte apoptosis in vitro. Urol. Int..

[187] Butler J.T., Abdelhamed S., Kurre P. (2018). Extracellular vesicles in the hematopoietic microenvironment. Haematologica.

[188] Nehrbas J., Butler J.T., Chen D.-W., Kurre P. (2020). Extracellular vesicles and chemotherapy resistance in the AML microenvironment. Front. Oncol..

[189] Patel G.K., Khan M.A., Bhardwaj A., Srivastava S.K., Zubair H., Patton M.C., Singh S., Singh A.P. (2017). Exosomes confer chemoresistance to pancreatic cancer cells by promoting ROS detoxification and miR-155-mediated suppression of key gemcitabine-metabolising enzyme, DCK. Br. J. Cancer.

[190] Wojtuszkiewicz A., Schuurhuis G.J., Kessler F.L., Piersma S.R., Knol J.C., Pham T.V., Jansen G., Musters R.J., van Meerloo J., Assaraf Y.G. (2016). Exosomes secreted by apoptosis-resistant acute myeloid leukemia (AML) blasts harbor regulatory network proteins potentially involved in antagonism of apoptosis. Mol. Cell. Proteom..

[191] Bouvy C., Wannez A., Laloy J., Chatelain C., Dogné J.-M. (2017). Transfer of multidrug resistance among acute myeloid leukemia cells via extracellular vesicles and their microRNA cargo. Leuk. Res..

[192] Daver N., Schlenk R.F., Russell N.H., Levis M.J. (2019). Targeting FLT3 mutations in AML: Review of current knowledge and evidence. Leukemia.

[193] Melo S.A., Luecke L.B., Kahlert C., Fernandez A.F., Gammon S.T., Kaye J., LeBleu V.S., Mittendorf E.A., Weitz J., Rahbari N. (2015). Glypican-1 identifies cancer exosomes and detects early pancreatic cancer. Nature.

[194] Costa-Silva B., Aiello N.M., Ocean A.J., Singh S., Zhang H., Thakur B.K., Becker A., Hoshino A., Mark M.T., Molina H. (2015). Pancreatic cancer exosomes initiate pre-metastatic niche formation in the liver. Nat. Cell Biol..

[195] Hu Y., Yan C., Mu L., Huang K., Li X., Tao D., Wu Y., Qin J. (2015). Fibroblast-derived exosomes contribute to chemoresistance through priming cancer stem cells in colorectal cancer. PLoS ONE.

[196] Qin X., Guo H., Wang X., Zhu X., Yan M., Wang X., Xu Q., Shi J., Lu E., Chen W. (2019). Exosomal miR-196a derived from cancer-associated fibroblasts confers cisplatin resistance in head and neck cancer through targeting CDKN1B and ING5. Genome Biol..

[197] Yeung C.L.A., Co N.-N., Tsuruga T., Yeung T.-L., Kwan S.-Y., Leung C.S., Li Y., Lu E.S., Kwan K., Wong K.-K. (2016). Exosomal transfer of stroma-derived miR21 confers paclitaxel resistance in ovarian cancer cells through targeting APAF1. Nat. Commun..

[198] Challagundla K.B., Wise P.M., Neviani P., Chava H., Murtadha M., Xu T., Kennedy R., Ivan C., Zhang X., Vannini I. (2015). Exosome-mediated transfer of microRNAs within the tumor microenvironment and neuroblastoma resistance to chemotherapy. JNCI J. Natl. Cancer Inst..

[199] Mc Namee N., O’Driscoll L. (2018). Extracellular vesicles and anti-cancer drug resistance. Biochim. Biophys. Acta Rev. Cancer.

[200] Lopes-Rodrigues V., Di Luca A., Mleczko J., Meleady P., Henry M., Pesic M., Cabrera D., van Liempd S., Lima R.T., O’Connor R. (2017). Identification of the metabolic alterations associated with the multidrug resistant phenotype in cancer and their intercellular transfer mediated by extracellular vesicles. Sci. Rep..

[201] Wang X., Zhang H., Chen X. (2019). Drug resistance and combating drug resistance in cancer. Cancer Drug Resist..

[202] Ye Q., Liu K., Shen Q., Li Q., Hao J., Han F., Jiang R.-W. (2019). Reversal of multidrug resistance in cancer by multi-functional flavonoids. Front. Oncol..

[203] Corcoran C., Rani S., O’Brien K., O’Neill A., Prencipe M., Sheikh R., Webb G., McDermott R., Watson W., Crown J. (2012). Docetaxel-resistance in prostate cancer: Evaluating associated phenotypic changes and potential for resistance transfer via exosomes. PLoS ONE.

[204] Rahman M., Hasan M.R. (2015). Cancer metabolism and drug resistance. Metabolites.

[205] Castro I., Xavier C., Vasconcelos M. (2017). Is P-glycoprotein relevant for the release of microvesicles by tumor cells?: PS212. Porto Biomed. J..

[206] Fontana F., Carollo E., Melling G.E., Carter D.R. (2021). Extracellular Vesicles: Emerging Modulators of Cancer Drug Resistance. Cancers.

[207] Wang X., Qiao D., Chen L., Xu M., Chen S., Huang L., Wang F., Chen Z., Cai J., Fu L. (2019). Chemotherapeutic drugs stimulate the release and recycling of extracellular vesicles to assist cancer cells in developing an urgent chemoresistance. Mol. Cancer.

[208] Levchenko A., Mehta B.M., Niu X., Kang G., Villafania L., Way D., Polycarpe D., Sadelain M., Larson S.M. (2005). Intercellular transfer of P-glycoprotein mediates acquired multidrug resistance in tumor cells. Proc. Natl. Acad. Sci. USA.

[209] Bebawy M., Combes V., Lee E., Jaiswal R., Gong J., Bonhoure A., Grau G. (2009). Membrane microparticles mediate transfer of P-glycoprotein to drug sensitive cancer cells. Leukemia.

[210] Chen W.-X., Cai Y.-Q., Lv M.-M., Chen L., Zhong S.-L., Ma T.-F., Zhao J.-H., Tang J.-H. (2014). Exosomes from docetaxel-resistant breast cancer cells alter chemosensitivity by delivering microRNAs. Tumor Biol..

[211] Yu S., Wei Y., Xu Y., Zhang Y., Li J., Zhang J. (2016). Extracellular vesicles in breast cancer drug resistance and their clinical application. Tumor Biol..

[212] Lopes-Rodrigues V., Di Luca A., Sousa D., Seca H., Meleady P., Henry M., Lima R.T., O’Connor R., Vasconcelos M.H. (2016). Multidrug resistant tumour cells shed more microvesicle-like EVs and less exosomes than their drug-sensitive counterpart cells. Biochim. Biophys. Acta Gen. Subj..

[213] Kwok H.-H., Ning Z., Chong P.W.-C., Wan T.S.-K., Ng M.H.-L., Ho G.Y., Ip M.S.-M., Lam D.C.-L. (2019). Transfer of extracellular vesicle-associated-RNAs induces drug resistance in ALK-translocated lung adenocarcinoma. Cancers.

[214] Yan X., Yin J., Yao H., Mao N., Yang Y., Pan L. (2010). Increased expression of annexin A3 is a mechanism of platinum resistance in ovarian cancer. Cancer Res..

[215] Ciravolo V., Huber V., Ghedini G.C., Venturelli E., Bianchi F., Campiglio M., Morelli D., Villa A., Mina P.D., Menard S. (2012). Potential role of HER2-overexpressing exosomes in countering trastuzumab-based therapy. J. Cell. Physiol..

[216] Mikamori M., Yamada D., Eguchi H., Hasegawa S., Kishimoto T., Tomimaru Y., Asaoka T., Noda T., Wada H., Kawamoto K. (2017). MicroRNA-155 controls exosome synthesis and promotes gemcitabine resistance in pancreatic ductal adenocarcinoma. Sci. Rep..

[217] Singh R.R., Reindl K.M. (2021). Glutathione S-Transferases in Cancer. Antioxidants (Basel).

[218] Zhang Q., Liu R.X., Chan K.W., Hu J., Zhang J., Wei L., Tan H., Yang X., Liu H. (2019). Exosomal transfer of p-STAT3 promotes acquired 5-FU resistance in colorectal cancer cells. J. Exp. Clin. Cancer Res. CR.

[219] Dong Y., Pan Q., Jiang L., Chen Z., Zhang F., Liu Y., Xing H., Shi M., Li J., Li X. (2014). Tumor endothelial expression of P-glycoprotein upon microvesicular transfer of TrpC5 derived from adriamycin-resistant breast cancer cells. Biochem. Biophys. Res. Commun..

[220] Nanayakkara A.K., Follit C.A., Chen G., Williams N.S., Vogel P.D., Wise J.G. (2018). Targeted inhibitors of P-glycoprotein increase chemotherapeutic-induced mortality of multidrug resistant tumor cells. Sci Rep.

[221] Hoskin V., Ghaffari A., Elliott B.E. (2019). Ezrin, more than a metastatic detERMinant?. Oncotarget.

[222] Yano K., Okabe C., Fujii K., Kato Y., Ogihara T. (2020). Regulation of breast cancer resistance protein and P-glycoprotein by ezrin, radixin and moesin in lung, intestinal and renal cancer cell lines. J. Pharm. Pharmacol..

[223] Pokharel D., Padula M.P., Lu J.F., Jaiswal R., Djordjevic S.P., Bebawy M. (2016). The Role of CD44 and ERM Proteins in Expression and Functionality of P-glycoprotein in Breast Cancer Cells. Molecules.

[224] Jiang Q.H., Wang A.X., Chen Y. (2014). Radixin enhances colon cancer cell invasion by increasing MMP-7 production via Rac1-ERK pathway. Sci. World J..

[225] Cho S.H., Park Y.S., Kim H.J., Kim C.H., Lim S.W., Huh J.W., Lee J.H., Kim H.R. (2012). CD44 enhances the epithelial-mesenchymal transition in association with colon cancer invasion. Int. J. Oncol..

[226] Szatanek R., Baj-Krzyworzeka M. (2021). CD44 and Tumor-Derived Extracellular Vesicles (TEVs). Possible Gateway to Cancer Metastasis. Int. J. Mol. Sci..

[227] Whitlock B.D., Leslie E.M. (2020). Efflux transporters in anti-cancer drug resistance: Molecular and functional identification and characterization of multidrug resistance proteins (MRPs/ABCCs). Drug Efflux Pumps in Cancer Resistance Pathways: From Molecular Recognition and Characterization to Possible Inhibition Strategies in Chemotherapy.

[228] Munoz M., Henderson M., Haber M., Norris M. (2007). Role of the MRP1/ABCC1 multidrug transporter protein in cancer. IUBMB Life.

[229] Goler-Baron V., Assaraf Y.G. (2011). Structure and function of ABCG2-rich extracellular vesicles mediating multidrug resistance. PLoS ONE.

[230] Fukumori T., Kanayama H.O., Raz A. (2007). The role of galectin-3 in cancer drug resistance. Drug Resist. Updates Rev. Comment. Antimicrob. Anticancer. Chemother..

[231] Tonissen K.F., Poulsen S.-A. (2021). Carbonic anhydrase XII inhibition overcomes P-glycoprotein-mediated drug resistance: A potential new combination therapy in cancer. Cancer Drug Resist..

[232] Ning K., Wang T., Sun X., Zhang P., Chen Y., Jin J., Hua D. (2017). UCH-L1-containing exosomes mediate chemotherapeutic resistance transfer in breast cancer. J. Surg. Oncol..

[233] Gong J., Luk F., Jaiswal R., George A.M., Grau G.E.R., Bebawy M. (2013). Microparticle drug sequestration provides a parallel pathway in the acquisition of cancer drug resistance. Eur. J. Pharmacol..

[234] Torreggiani E., Roncuzzi L., Perut F., Zini N., Baldini N. (2016). Multimodal transfer of MDR by exosomes in human osteosarcoma. Int. J. Oncol..

[235] Jaiswal R., Luk F., Dalla P.V., Grau G.E.R., Bebawy M. (2013). Breast cancer-derived microparticles display tissue selectivity in the transfer of resistance proteins to cells. PLoS ONE.

[236] Hongmei Z. (2012). Extrinsic and Intrinsic Apoptosis Signal Pathway Review.

[237] Gregory C.D., Dransfield I. (2018). Apoptotic tumor cell-derived extracellular vesicles as important regulators of the onco-regenerative niche. Front. Immunol..

[238] Westhoff M.-A., Marschall N., Debatin K.-M. (2016). Novel approaches to apoptosis-inducing therapies. Apoptosis Cancer Pathog. Anti-Cancer Ther..

[239] Jiang N., Dai Q., Su X., Fu J., Feng X., Peng J. (2020). Role of PI3K/AKT pathway in cancer: The framework of malignant behavior. Mol. Biol. Rep..

[240] Dong H., Wang W., Chen R., Zhang Y., Zou K., Ye M., He X., Zhang F., Han J. (2018). Exosome-mediated transfer of lncRNA-SNHG14 promotes trastuzumab chemoresistance in breast cancer. Int. J. Oncol..

[241] Khoo X.-H., Paterson I.C., Goh B.-H., Lee W.-L. (2019). Cisplatin-resistance in oral squamous cell carcinoma: Regulation by tumor cell-derived extracellular vesicles. Cancers.

[242] Brzozowski J.S., Jankowski H., Bond D.R., McCague S.B., Munro B.R., Predebon M.J., Scarlett C.J., Skelding K.A., Weidenhofer J. (2018). Lipidomic profiling of extracellular vesicles derived from prostate and prostate cancer cell lines. Lipids Health Dis..

[243] Soekmadji C., Nelson C.C. (2015). The emerging role of extracellular vesicle-mediated drug resistance in cancers: Implications in advanced prostate cancer. BioMed Res. Int..

[244] Trajkovic K., Hsu C., Chiantia S., Rajendran L., Wenzel D., Wieland F., Schwille P., Brügger B., Simons M. (2008). Ceramide triggers budding of exosome vesicles into multivesicular endosomes. Science.

[245] Faict S., Oudaert I., D’Auria L., Dehairs J., Maes K., Vlummens P., De Veirman K., De Bruyne E., Fostier K., Vande Broek I. (2019). The transfer of sphingomyelinase contributes to drug resistance in multiple myeloma. Cancers.

[246] Srivastava A., Amreddy N., Pareek V., Chinnappan M., Ahmed R., Mehta M., Razaq M., Munshi A., Ramesh R. (2020). Progress in extracellular vesicle biology and their application in cancer medicine. Wiley Interdiscip. Rev. Nanomed. Nanobiotechnol..

[247] Eichelser C., Stückrath I., Müller V., Milde-Langosch K., Wikman H., Pantel K., Schwarzenbach H. (2014). Increased serum levels of circulating exosomal microRNA-373 in receptor-negative breast cancer patients. Oncotarget.

[248] Lucien F., Lac V., Billadeau D.D., Borgida A., Gallinger S., Leong H.S. (2019). Glypican-1 and glycoprotein 2 bearing extracellular vesicles do not discern pancreatic cancer from benign pancreatic diseases. Oncotarget.

[249] Vader P., Breakefield X.O., Wood M.J. (2014). Extracellular vesicles: Emerging targets for cancer therapy. Trends Mol. Med..

[250] Chen Y., Wang L., Zhu Y., Chen Z., Qi X., Jin L., Jin J., Hua D., Ma X. (2015). Breast cancer resistance protein (BCRP)-containing circulating microvesicles contribute to chemoresistance in breast cancer. Oncol. Lett..

[251] Corcoran C., Rani S., O’Driscoll L. (2014). miR-34a is an intracellular and exosomal predictive biomarker for response to docetaxel with clinical relevance to prostate cancer progression. Prostate.

[252] Li L., Li C., Wang S., Wang Z., Jiang J., Wang W., Li X., Chen J., Liu K., Li C. (2016). Exosomes derived from hypoxic oral squamous cell carcinoma cells deliver miR-21 to normoxic cells to elicit a prometastatic phenotype. Cancer Res..

[253] Shukuya T., Ghai V., Amann J.M., Okimoto T., Shilo K., Kim T.-K., Wang K., Carbone D.P. (2020). Circulating MicroRNAs and Extracellular Vesicle–Containing MicroRNAs as Response Biomarkers of Anti–programmed Cell Death Protein 1 or Programmed Death-Ligand 1 Therapy in NSCLC. J. Thorac. Oncol..

[254] Sidransky D. (2002). Emerging molecular markers of cancer. Nat. Rev. Cancer.

[255] Tutrone R., Donovan M.J., Torkler P., Tadigotla V., McLain T., Noerholm M., Skog J., McKiernan J. (2020). Clinical utility of the exosome based ExoDx Prostate (IntelliScore) EPI test in men presenting for initial Biopsy with a PSA 2–10 ng/mL. Prostate Cancer Prostatic Dis..

[256] Lin S.-Y., Chang C.-H., Wu H.-C., Lin C.-C., Chang K.-P., Yang C.-R., Huang C.-P., Hsu W.-H., Chang C.-T., Chen C.-J. (2016). Proteome profiling of urinary exosomes identifies alpha 1-antitrypsin and H2B1K as diagnostic and prognostic biomarkers for urothelial carcinoma. Sci. Rep..

[257] Trino S., Lamorte D. (2021). Clinical relevance of extracellular vesicles in hematological neoplasms: From liquid biopsy to cell biopsy. Leukemia.

[258] Pang B., Zhu Y., Ni J., Thompson J., Malouf D., Bucci J., Graham P., Li Y. (2020). Extracellular vesicles: The next generation of biomarkers for liquid biopsy-based prostate cancer diagnosis. Theranostics.

[259] Tavoosidana G., Ronquist G., Darmanis S., Yan J., Carlsson L., Wu D., Conze T., Ek P., Semjonow A., Eltze E. (2011). Multiple recognition assay reveals prostasomes as promising plasma biomarkers for prostate cancer. Proc. Natl. Acad. Sci. USA.

[260] Rabinowits G., Gerçel-Taylor C., Day J.M., Taylor D.D., Kloecker G.H. (2009). Exosomal microRNA: A diagnostic marker for lung cancer. Clin. Lung Cancer.

[261] Chang L., Ni J., Zhu Y., Pang B., Graham P., Zhang H., Li Y. (2019). Liquid biopsy in ovarian cancer: Recent advances in circulating extracellular vesicle detection for early diagnosis and monitoring progression. Theranostics.

[262] Mathai R.A., Vidya R.V.S., Reddy B.S., Thomas L., Udupa K., Kolesar J., Rao M. (2019). Potential Utility of Liquid Biopsy as a Diagnostic and Prognostic Tool for the Assessment of Solid Tumors: Implications in the Precision Oncology. J. Clin. Med..

[263] Hoshino A., Kim H.S., Bojmar L., Gyan K.E., Cioffi M., Hernandez J., Zambirinis C.P., Rodrigues G., Molina H., Heissel S. (2020). Extracellular Vesicle and Particle Biomarkers Define Multiple Human Cancers. Cell.

[264] Wu S., Zhu W., Thompson P., Hannun Y.A. (2018). Evaluating intrinsic and non-intrinsic cancer risk factors. Nat. Commun..

[265] Kosaka N., Iguchi H., Ochiya T. (2010). Circulating microRNA in body fluid: A new potential biomarker for cancer diagnosis and prognosis. Cancer Sci..

[266] Cabeza L., Perazzoli G., Peña M., Cepero A., Luque C., Melguizo C., Prados J. (2020). Cancer therapy based on extracellular vesicles as drug delivery vehicles. J. Control Release.

[267] Cao X.-H., Liang M.-X., Wu Y., Yang K., Tang J.-H., Zhang W. (2021). Extracellular vesicles as drug vectors for precise cancer treatment. Nanomedicine.

[268] Agrawal A.K., Aqil F., Jeyabalan J., Spencer W.A., Beck J., Gachuki B.W., Alhakeem S.S., Oben K., Munagala R., Bondada S. (2017). Milk-derived exosomes for oral delivery of paclitaxel. Nanomed. Nanotechnol. Biol. Med..

[269] Van der Meel R., Fens M.H., Vader P., Van Solinge W.W., Eniola-Adefeso O., Schiffelers R.M. (2014). Extracellular vesicles as drug delivery systems: Lessons from the liposome field. J. Control Release.

[270] Lee Y., Thompson D. (2017). Stimuli-responsive liposomes for drug delivery. Wiley Interdiscip. Rev. Nanomed. Nanobiotechnol..

[271] Khawar M.B., Abbasi M.H., Siddique Z., Arif A., Sheikh N. (2019). An update on novel therapeutic warfronts of extracellular vesicles (EVs) in cancer treatment: Where we are standing right now and where to go in the future. Oxidative Med. Cell. Longev..

[272] Gaurav I., Thakur A., Iyaswamy A., Wang X., Chen X., Yang Z. (2021). Factors Affecting Extracellular Vesicles Based Drug Delivery Systems. Molecules.

[273] Murphy D.E., de Jong O.G., Brouwer M., Wood M.J., Lavieu G., Schiffelers R.M., Vader P. (2019). Extracellular vesicle-based therapeutics: Natural versus engineered targeting and trafficking. Exp. Mol. Med..

[274] Springer A.D., Dowdy S.F. (2018). GalNAc-siRNA conjugates: Leading the way for delivery of RNAi therapeutics. Nucleic Acid Ther..

[275] Lamichhane T.N., Jeyaram A., Patel D.B., Parajuli B., Livingston N.K., Arumugasaamy N., Schardt J.S., Jay S.M. (2016). Oncogene knockdown via active loading of small RNAs into extracellular vesicles by sonication. Cell. Mol. Bioeng..

[276] Tian H., Li W. (2017). Dendritic cell-derived exosomes for cancer immunotherapy: Hope and challenges. Ann. Transl. Med..

[277] Whiteside T.L. (2017). Exosomes in cancer: Another mechanism of tumor-induced immune suppression. Tumor Immune Microenviron. Cancer Progress. Cancer Ther..

[278] Yang X., Chen J., Wang N., Liu Z., Li Y. (2018). Clinical use of dendritic cell-derived exosomes for hepatocellular carcinoma immunotherapy: How far we are?. J. Hepatol..

[279] Luo R., Liu M., Tan T., Yang Q., Wang Y., Men L., Zhao L., Zhang H., Wang S., Xie T. (2021). Emerging Significance and Therapeutic Potential of Extracellular vesicles. Int. J. Biol. Sci..

[280] Campos J.H., Soares R.P., Ribeiro K., Cronemberger Andrade A., Batista W.L., Torrecilhas A.C. (2015). Extracellular vesicles: Role in inflammatory responses and potential uses in vaccination in cancer and infectious diseases. J. Immunol. Res..

[281] Whiteside T. (2017). Exosomes carrying immunoinhibitory proteins and their role in cancer. Clin. Exp. Immunol..

[282] Koppers-Lalic D., Hogenboom M.M., Middeldorp J.M., Pegtel D.M. (2013). Virus-modified exosomes for targeted RNA delivery; A new approach in nanomedicine. Adv. Drug Deliv. Rev..

[283] Wirth T., Ylä-Herttuala S. (2014). Gene therapy used in cancer treatment. Biomedicines.

[284] Mizrak A., Bolukbasi M.F., Ozdener G.B., Brenner G.J., Madlener S., Erkan E.P., Ströbel T., Breakefield X.O., Saydam O. (2013). Genetically engineered microvesicles carrying suicide mRNA/protein inhibit schwannoma tumor growth. Mol. Ther. J. Am. Soc. Gene Ther..

[285] Erkan E.P., Senfter D., Madlener S., Jungwirth G., Ströbel T., Saydam N., Saydam O. (2017). Extracellular vesicle-mediated suicide mRNA/protein delivery inhibits glioblastoma tumor growth in vivo. Cancer Gene Ther..

[286] Alexander M., Hu R., Runtsch M.C., Kagele D.A., Mosbruger T.L., Tolmachova T., Seabra M.C., Round J.L., Ward D.M., O’Connell R.M. (2015). Exosome-delivered microRNAs modulate the inflammatory response to endotoxin. Nat. Commun..

[287] Usman W.M., Pham T.C., Kwok Y.Y., Vu L.T., Ma V., Peng B., Chan Y.S., Wei L., Chin S.M., Azad A. (2018). Efficient RNA drug delivery using red blood cell extracellular vesicles. Nat. Commun..

[288] Caforio M., Sorino C., Bertaina V., Pitisci A., Battafarano G., Del Fattore A., Fanciulli M., Folgiero V., Locatelli F. (2019). PB1649 Exosomes-Mediated Delivery of RNA Oligos Directed to Che-1/Aatf Impairs Bcp-All Vitality. HemaSphere.

[289] Lin Y., Wu J., Gu W., Huang Y., Tong Z., Huang L., Tan J. (2018). Exosome-Liposome Hybrid Nanoparticles Deliver CRISPR/Cas9 System in MSCs. Adv. Sci..

[290] Kim S.M., Yang Y., Oh S.J., Hong Y., Seo M., Jang M. (2017). Cancer-derived exosomes as a delivery platform of CRISPR/Cas9 confer cancer cell tropism-dependent targeting. J. Control. Release.

[291] Börger V., Weiss D.J., Anderson J.D., Borràs F.E., Bussolati B., Carter D.R.F., Dominici M., Falcón-Pérez J.M., Gimona M., Hill A.F. (2020). International Society for Extracellular Vesicles and International Society for Cell and Gene Therapy statement on extracellular vesicles from mesenchymal stromal cells and other cells: Considerations for potential therapeutic agents to suppress coronavirus disease-19. Cytotherapy.

[292] Wang J.H., Forterre A.V., Zhao J., Frimannsson D.O., Delcayre A., Antes T.J., Efron B., Jeffrey S.S., Pegram M.D., Matin A.C. (2018). Anti-HER2 scFv-Directed Extracellular Vesicle-Mediated mRNA-Based Gene Delivery Inhibits Growth of HER2-Positive Human Breast Tumor Xenografts by Prodrug Activation. Mol. Cancer Ther..

[293] Massaro C., Sgueglia G., Frattolillo V., Baglio S.R., Altucci L. (2020). Extracellular Vesicle-Based Nucleic Acid Delivery: Current Advances and Future Perspectives in Cancer Therapeutic Strategies. Pharmaceutics.

[294] Lewis N.D., Sia C.L., Kirwin K., Haupt S. (2021). Exosome Surface Display of IL12 Results in Tumor-Retained Pharmacology with Superior Potency and Limited Systemic Exposure Compared with Recombinant IL12. Mol. Cancer Ther..

[295] Lewis N., Sia C.L., Kirwin K., Haupt S., Mahimkar G., Zi T., Xu K., Dooley K., Jang S.C., Choi B. (2020). 709 Exosome surface display of IL-12 results in tumor-retained pharmacology with superior potency and limited systemic exposure. BMJ Spec. J..

[296] codiakbio.com. https://ir.codiakbio.com/news-releases/news-release-details/codiak-initiates-patient-dosing-phase-12-clinical-trial.

[297] codiakbio.com. https://ir.codiakbio.com/node/6411/pdf.

[298] biospace.com. https://www.biospace.com/article/un-stranding-assets-with-evs/.

[299] omnispirant.com. https://www.omnispirant.com/.

[300] carminetherapeutics.com. https://www.carminetherapeutics.com/.

[301] Roefs M.T., Sluijter J.P., Vader P. (2020). Extracellular vesicle-associated proteins in tissue repair. Trends Cell Biol..

[302] Meng W., He C., Hao Y., Wang L., Li L., Zhu G. (2020). Prospects and challenges of extracellular vesicle-based drug delivery system: Considering cell source. Drug Deliv..

[303] Kennedy T.L., Russell A.J., Riley P. (2021). Experimental limitations of extracellular vesicle-based therapies for the treatment of myocardial infarction. Trends Cardiovasc. Med..

